# Synthesis of Ni-Co Metal–Organic Framework (Ni-Co MOF) Structures by High-Power, Continuous Laser-Induced Rapid Synthesis Method and Investigation of Their Morphological, Structural, Photophysical, and Electrical Properties

**DOI:** 10.3390/polym18161985

**Published:** 2026-08-14

**Authors:** Saliha Mutlu, Bülend Ortaç, Ali Karatutlu, Vildan Yılmaz, Süreyya Aydin Yüksel, Ahmet Hakan Yilmaz, Nergis Arsu, Sevil Savaskan Yilmaz

**Affiliations:** 1 Faculty of Science, Department of Chemistry, Karadeniz Technical University, Trabzon 61080, Turkey; salihamutlu@ktu.edu.tr; 2 Institute of Materials Science Nanotechnology and National Nanotechnology Research Center (UNAM), Bilkent University, Ankara 06800, Turkey; 3 Engineering Fundamental Sciences, Sivas University of Science and Technology, Sivas 58000, Turkey; ali@unam.bilkent.edu.tr; 4 Department of Physics, Davutpasa Campus, Yildiz Technical University, Istanbul 34220, Turkey; vyilmaz@yildiz.edu.tr (V.Y.); suaydin@yildiz.edu.tr (S.A.Y.); 5 Faculty of Science, Department of Physic, Karadeniz Technical University, Trabzon 61080, Turkey; hakany@ktu.edu.tr; 6 Department of Chemistry, Davutpasa Campus, Yildiz Technical University, Istanbul 34220, Turkey

**Keywords:** metal–organic frameworks, laser-induced synthesis, bimetallic MOF, electrochemical performance, polymeric film

## Abstract

Metal–organic bimetallic frameworks of Ni–Co, having metal content of 2:1 and 1:2 molar ratios, respectively, have been synthesized via a rapid laser method with a continuous-wave Nd:YVO_4_ laser (λ = 975 nm) under 88–90 °C in a DMF/H_2_O solution in 70 min. The structure, porosity, and photophysical, electrochemical, and dielectric characteristics of the frameworks and their reduced graphene oxide (rGO) composites in the form of powders and UV-cured PEGMEA/PEGDA films have been investigated. Framework Ni2Co1MOF demonstrated a BET surface area equal to 88.3 m^2^ g^−1^ and a total pore volume of 0.022 cm^3^ g^−1^, whereas framework Ni1Co2MOF exhibited a BET surface area of 52.5 m^2^ g^−1^ and a total pore volume of 0.016 cm^3^ g^−1^. The incorporation of rGO from 1 to 10 wt.% into the framework changed the charge transport and polarization properties of the materials. The electrochemical investigations of the 10 wt.% rGO-Ni1Co2MOF composite in 0.5 M HCl demonstrated a specific capacitance of 32.3 F g^−1^ at 10 mV s^−1^, and it preserved 98% of the electrochemical response after 400 cycles, in comparison with 96% for the 10 wt.% rGO-Ni2Co1MOF. The electrochemical responses consisted of both diffusion-controlled ion transport and pseudocapacitance. The introduction of rGO in 1 to 10 wt.% in the polymer composite improved the conductivity and Maxwell–Wagner–Sillars interface polarization at low frequencies in the case of low rGO concentrations, whereas overly high rGO content led to aggregation and the decreased influence of the conductive phase. The main contribution of the present work is the fast sub-100 °C synthesis approach for compositionally tunable Ni-Co frameworks/rGO materials and the relationships between the metal ratio, porous structure, interfacial charge transport, and dielectric response.

## 1. Introduction

Metal–organic frameworks (MOFs), which are involved in various applications across the fields of science and technology, are crystalline and porous structures formed through the coordination of metal ions/clusters and organic bridging ligands via chemical bonds. In MOFs, the strong bonding of inorganic and organic units to form periodic network structures gives rise to large surface areas and high porosity within their structures [1]. Since their announcement in 1995 by Omar Yaghi and his team, MOFs have continued to develop rapidly, and studies on these materials have persisted to the present day; more than 90,000 MOF structures have been reported, with this number steadily increasing [2]. The organic building blocks are generally ditopic or multitopic carboxylates, as well as other similarly charged molecules, which form robust crystalline structures with characteristic porosity when coordinated to metal-containing units. Through the careful selection of metal ions and organic ligands, the design of desired structural, magnetic, electrical, optical, and catalytic properties has become possible, enabling MOFs to exhibit tailored functions and performance in different fields [3].

The main methods used for MOF structure synthesis include solvothermal, microwave, ultrasonic, electrochemical, mechanical, ionic fluid, microfluidic, and dry-gel synthesis approaches [4]. At present, MOFs cannot be produced industrially. It should be understood that there are several criteria that need to be taken into account to start mass production. This includes inexpensive raw materials, low temperatures and pressures, high yields, purification to eliminate significant amounts of impurities, and low consumption of solvents [5]. Most MOFs are obtained by means of solvothermal methods, with high temperatures and extended periods of reaction. Thus, the industrial approach shows inefficiency, excessive time consumption, and high energy costs. Over the past few years, many reports have confirmed that materials based on graphene, which are remarkable in terms of their electrochemical and mechanical properties, can be mixed with MOFs, providing not only high electrical conductivity and mechanical strength but also preventing aggregation between layers of graphene. Graphene-based composites are widely used because of their low cost, mechanical strength, large active surface area, conductivity, and further modification potential. For example, unlike rGO, graphene oxide (GO) demonstrates poor behavior in terms of retaining its physical and chemical properties (high thermal and electrical conductivity), so it requires a reduction process to improve them, which is achievable by means of a reducing agent (hydrazine hydrate), eliminating the functional groups of oxygen [6]. Thanks to the unique sp^2^ domains, rGO can provide an adequate solution to the problem of the weak coordination binding of metals with organic ligands, thereby increasing the scope for the practical use of MOFs [7]. In particular, rGO has received much attention recently due to the combination of unique physical and chemical properties, an enormous surface area, high electrical conductivity, good biocompatibility, chemical stability, and excellent bonding properties [8]. Moreover, it has been established that the inclusion of rGO layers in crystalline MOFs can lead to increased conductivity, yielding MOFs suitable for chemically resistant gas sensing. Bimetallic MOFs can outperform monometallic MOFs in a wide range of applications due to the synergic effect between two metals. The replacement of part of the original metal with a second metal provides an effective way to produce bimetallic MOFs. In bimetallic MOFs, two metal centers in one frame can control charge conduction, hence increasing the conductivity by regulating the ratio of metals [9,10]. Generally, MOFs of nickel have high capacitance and a disordered shape [11], while Co-based MOFs possess a regular and stable morphology and a low capacity [12]. Since both nickel and cobalt ions belong to divalent state metals with the same atomic number and almost identical ionic radii, nickel and cobalt ions can bind with one ligand to create a bimetallic MOF with minimal disordering in the crystal lattice. Moreover, some studies have revealed that it is possible to create homogeneous bimetallic MOFs, where two different metal ions can bind with one ligand without the formation of two separate monometallic MOFs. The creation of bimetallic MOFs is considered a novel and efficient strategy for the improvement of the characteristics of frameworks [13].

**Figure 1:**
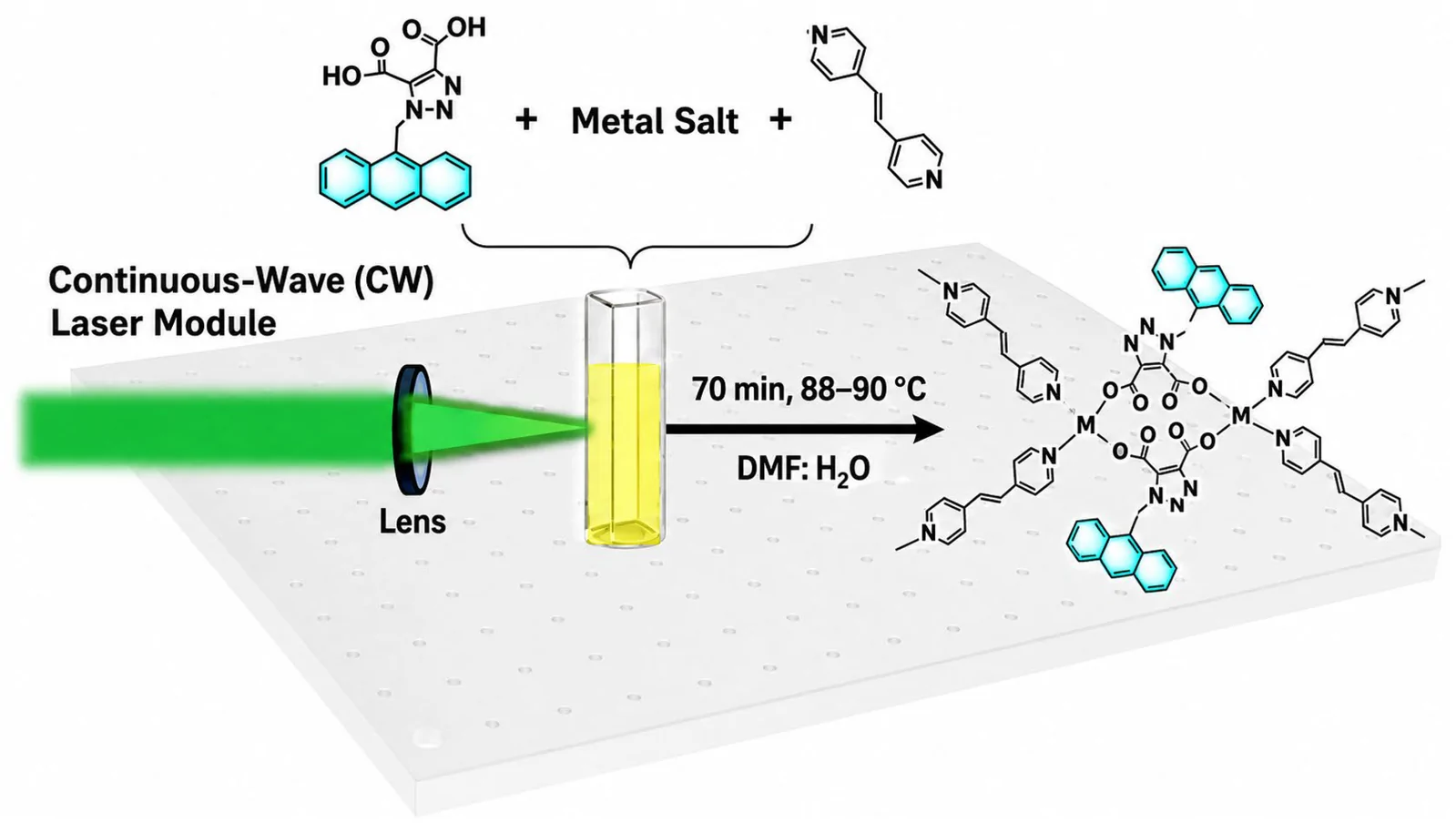
Schematic illustration of the laser-induced rapid synthesis (LIRS) process used for the preparation of Ni-Co MOF (2:1–1:2).

In our study, the optimal synthesis method for obtaining MOFs in a very short time, such as one hour, was proposed, and novel Ni/Co bimetallic MOFs (1:2 and 2:1) were successfully obtained via the LIRS synthesis method. Figure 1 is a schematic representation of the Ni-Co MOF synthesis procedure via the LIRS procedure. We suggest that the LIRS method could be a novel technique in comparison with classic methods, focused on effective short-term production and the electrochemical performance of MOFs. In addition, rGO composites in solutions were investigated. Moreover, rGO/MOF complexes obtained through the LIRS synthesis method were mixed with polymer films of PEGMEA/PEGDA, and their capacitance and conductivity characteristics were studied.

Firstly, Zn-based MOFs were successfully obtained by means of our proposed high-power method LIRS, in an extremely short production time (about one hour), in contrast to all other known MOF production methods with comparable morphologies [14].

Composites with MOFs have recently become highly popular among researchers. Conventional techniques are more appropriate for MOF composites prepared by post-modification and in situ synthesis methods [15]. Another method that can be used to synthesize numerous composites involving MOFs is laser-assisted synthesis, which has been widely studied in the literature over the last decade [16]. Composites make MOFs more valuable, since they bring new qualities or improve already present features of MOFs. Graphene is a two-dimensional material whose structure resembles a honeycomb, and it has properties such as high conductivity, electrocatalytic activity, a large surface area, and high biocompatibility. The use of composites containing graphene or GO increases the rates of electron transfer processes. Numerous researches have indicated that MOF/graphene or MOF/GO composites are perfect sensors for hydrogen peroxide, dopamine, paracetamol, aptasensors, monensin, and other analytes in human serum [17].

## 2. Materials and Methods

### 2.1. Chemicals

All reagents were commercially available from Sigma-Aldrich/Merck (St. Louis, MO, USA) and Alfa Aesar (Kandel, Germany) and were used without further purification. 1-(Anthracene-9-methyl)-1H-1,2,3-triazol-4,5-dicarboxylic acid (H_2_L) was synthesized according to the literature [18]. 9-(hydroxymethyl)anthracene (97%, Sigma-Aldric), phosphorus tribromide (99%, Sigma-Aldrich), sodium carbonate (Na_2_CO_3_, Merck), magnesium sulfate (anhydrous MgSO_4_, Merck), 9-(chloromethyl)anthracene (98%, Alfa Aesar), sodium azide (>99.5%, NaN_3_, Sigma-Aldrich), acetylene dicarboxylic acid (Sigma-Aldrich), 1,2-bis(4-pyridyl)ethene (bpe, Sigma-Aldrich), DMF (GC, Sigma-Aldrich), acetone (GC grade, Sigma-Aldrich), ethyl acetate (Sigma), Zn(NO_3_)_2_·6H_2_O (Sigma-Aldrich), NiCl_2_·6H_2_O (Sigma-Aldrich), CoCl_2_·6H_2_O (Sigma-Aldrich), Fe(Cl)_2_·4H_2_O (Sigma-Aldrich), poly (ethylene glycol) methyl ether acrylate (PEGMEA, Mn = 550, Sigma Aldrich), poly (ethylene glycol) diacrylate (PEGDA, Mn = 700, Sigma Aldrich), silver nitrate (>99.9%, Sigma-Aldrich), potassium hydroxide (Sigma-Aldrich), and hydrochloric acid (Sigma-Aldrich) were also used.

### 2.2. Instruments and Methods Used

NMR spectra of H_2_L were recorded by employing an Agilent VNMRS 500 (Santa Clara, CA, USA) operating at 500 MHz in DMSO d6 solution. Laser-assisted MOF synthesis was conducted employing a water-cooled diode laser with a fiber-coupled CW laser module, LIMO GmbH (Dortmund, Germany), with output power of 20 W and a 975 nm wavelength. To obtain an accurately collimated beam on the production platform, two flat convex lenses were used to collineate the laser beam emerging from the fiber output. Spectroscopy measurements (including UV-VIS-NIR) were performed for the characterization of the produced materials utilizing a Varian Cary 5000 (Santa Clara, CA, USA). Fluorescence and fluorescence lifetime measurements of the products were conducted using a Horiba Jobin Yvon Fluoromax-P spectrofluorometer (Paris, France). Fluorescence imaging of the samples was performed with a Leica confocal microscope incorporating a broadband visible light source and a 20X dry objective. IR spectra were recorded by employing a Bruker VERTEX 70 spectrometer (Ankara, Turkey) equipped with a Platinum-ATR accessory for the range of 400–4000 cm^−1^. SEM analysis of the morphologies of MOFs and their composites was achieved via the FEI Quanta 200 F (Oregon, OR, USA) and ZEISS EVO LS 10 (Ankara, Turkey) devices. Elemental composition analysis of the MOFs was conducted using the Ametek EDAX energy-dispersive X-ray (EDX) (Tokyo, Japan) technique. X-ray diffraction data for MOFs and MOF composites were obtained with a Malvern Panalytical X’pert Pro multifunctional multipurpose X-ray diffractometer (Almelo, The Netherlands) with Cu (45 kV–40 mA) and Cu Ka1 radiation (λ = 1.54059 Å). The thermal properties of MOFs and MOF composites were studied through thermogravimetric analysis (TGA) with a SEIKO 6300 TGA device (Tokyo, Japan). Photoelectrons created due to electron ejection from the atoms on the MOF surface were studied by employing an electron analyzer for the investigation of electron binding energies. Electron binding energies were determined using an X-ray photoelectron spectrometer (XPS; Thermo Scientific K-Alpha, Waltham, MA, USA). The XPS spectrum was analyzed with the Thermo Scientific Avantage software (v5.9916). N_2_ adsorption–desorption measurements were conducted using the BET method on a Quantachrome instrument.

Electrochemical studies were performed using a portable electrochemical analyzer, the Emstat4s electrochemical analysis instrument (PalmSens, Houten, The Netherlands), and the data were analyzed using the PSTrace 5.9 software. The measurements were performed using a graphite working electrode with a diameter of 3 mm, a glassy carbon electrode (3 mm diameter), a Ag pseudo-reference electrode, and a platinum counter electrode. MOF/polymer composite films were deposited on glass slides at a thickness of 40 µm through the roll coating technique and were cured through a Primarc Mini-UV curing system. Four-probe probe measurements were carried out using a Keithley semiconductor parameter analyzer (Solon, OH, USA). Dielectric measurements were performed under ambient conditions with an HP 4192A impedance analyzer (Hewlett-Packard, Palo Alto, CA, USA) within the frequency range of 10–10^6^ Hz. The dielectric measurements of the composites were performed in a sandwich configuration using dielectric ITO electrodes through an HP 4192A impedance analyzer at room temperature (10–10^7^ Hz).

## 3. Results

### 3.1. Synthesis and Characterization Studies of NiCoMOF (2:1–1:2) by LIRS Method

**Figure 2:**
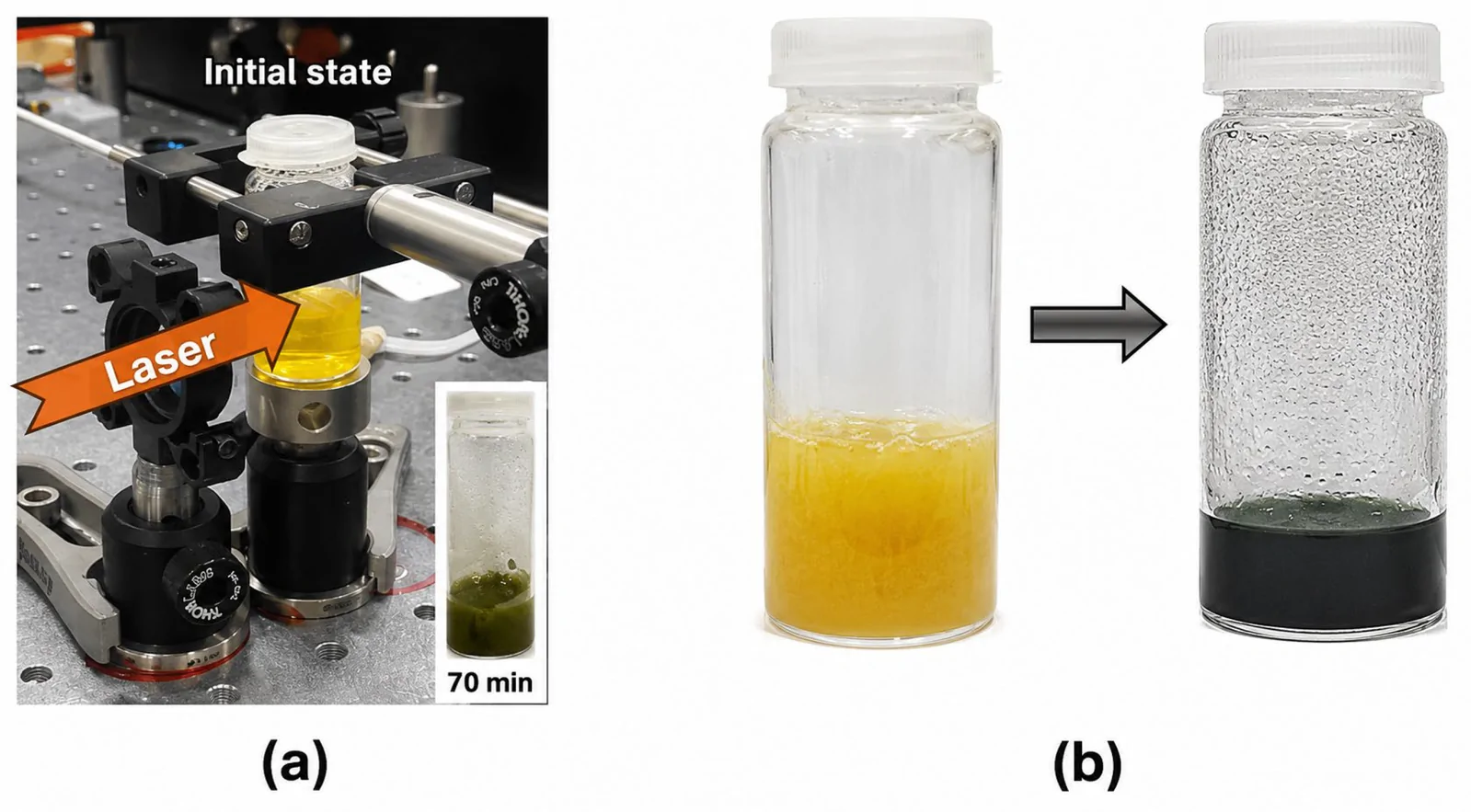
The synthesis of Ni2Co1MOF (**a**) and Ni1Co2MOF (**b**) by LIRS method.

Here, 0.022 mmol of NiCl_2_.6H_2_O, 0.011 mmol of CoCl_2_.6H_2_O, 0.033 mmol of H_2_L, and 0.066 mmol of bpe were dissolved in the DMF/H_2_O = 1:1 solvent mixture within 5 min. Dark-yellow-colored crystals of the MOF (Figure 2a) based on the cobalt metal ion were successfully prepared at 88–90 °C within 70 min in a continuous high-energy laser beam (975 nm). Crystals synthesized using DMF as a solvent three times were washed and dried in the oven at 60 °C overnight (90% yield).

Next, 0.011 mmol NiCl_2_.6H_2_O, 0.022 mmol CoCl_2_.6H_2_O (0.022 mmol), 0.033 mmol H_2_L, and 0.066 mmol bpe were combined in the DMF/H_2_O = 1:1 *v*/*v* solvent mixture for 5 min. Cobalt-containing MOFs were produced by irradiation with a continuous high-powered laser beam (975 nm) at 88–90 °C for 70 min. The obtained MOF crystals were purified three times with DMF and dried under a vacuum at 60 °C (yield: 85%). This process was carried out using the LIRS apparatus described in Figure 2b.

The direct preparation of bimetallic metal–organic framework (MOF) materials is commonly based on conventional hydrothermal and solvothermal techniques, where sealed autoclaves are applied along with high temperatures (>100 °C) and long reaction times ranging from several hours to even days. The optimization of different process parameters, including the solvent composition, pH, metal-to-ligand molar ratios, and temperature, is essential to obtain the required crystal structure and morphology. Therefore, energy-inefficient and lengthy synthesis methods constitute intrinsic disadvantages of the conventional liquid-phase synthesis of bimetallic MOFs. As such, the development of novel fast and energy-efficient strategies has become an important direction in the scalable production of advanced bimetallic MOFs [19,20,21]. In this respect, Ni-Co bimetallic MOFs have been successfully synthesized through the hydrothermal technique with high temperatures and extended reaction times.

It was possible to obtain bimetallic Ni-Co MOFs using the LIRS method within only 70 min at relatively low operating temperatures of 88–90 °C. This constitutes a great step ahead in comparison with the conventional hydrothermal and solvothermal techniques that need several hours of reaction at elevated temperatures. The energy savings in this process provide important advantages in terms of the energy efficiency and scalability of MOF material production.

### 3.2. Preparation of rGO/MOF Composites by In Situ Method

#### 3.2.1. Synthesis of Graphene Oxide (GO) and rGO

Graphene oxide (GO) was synthesized according to the modified Hummers method reported in the literature [22]. First, 2 g of graphite powder and 2 g of NaNO_3_ were dispersed in 50 mL of concentrated H_2_SO_4_. The mixture was cooled in an ice bath for 2 h. Then, the temperature was adjusted to 35 ± 5 °C, and 6 g of KMnO_4_ was gradually added while stirring continuously. The reaction was maintained at this temperature for an additional 2 h. Thereafter, 100 mL of deionized water was slowly added while keeping the reaction temperature below 90 °C. The mixture was further diluted with 200 mL of deionized water; then, 10 mL of H_2_O_2_ was added. After the suspension turned brownish yellow, the resulting solid was collected by centrifugation and washed repeatedly with deionized water until the pH reached 6.5–7.0. The obtained GO was then vacuum-dried at 80 °C. The rGO was prepared by dispersing 100 mg of GO in 100 mL of deionized water, followed by ultrasonication for 2 h. Subsequently, 1 mL of hydrazine hydrate was added, and the suspension was heated at 100 °C for 24 h in a water bath. The resulting black rGO precipitate was collected by filtration and thoroughly washed with 500 mL of deionized water and 500 mL of methanol (CH_3_OH) before use [23].

#### 3.2.2. Preparation of 1%, 3%, 5%, and 10% rGO/MOF Composites by In Situ Method

**Figure 3:**
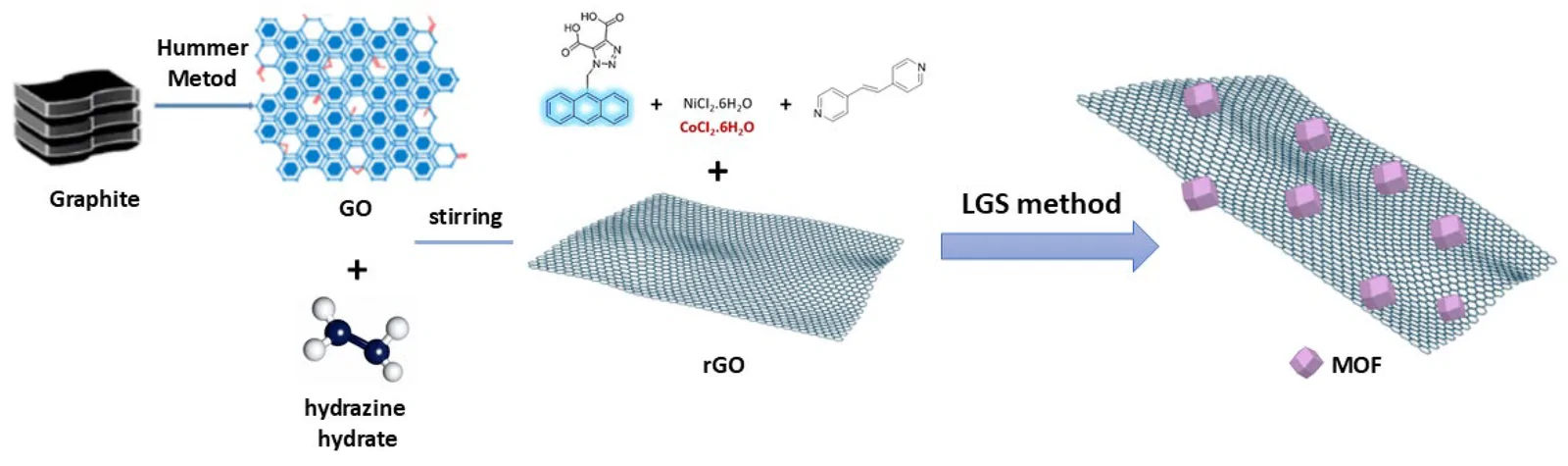
Schematic illustration of the preparation of rGO-containing MOF composites via the LIRS method.

MOF and rGO composites were prepared by the LIRS method. For the preparation of the composites, metal salt (0.033 mmol), H_2_L (0.033 mmol), and bpe (0.066 mmol) ligands were added to DMF/H_2_O (1:1 by volume) while continuously stirring. Then, rGO (1, 3, 5, and 10% by weight) was added to the solution and it was stirred for 2 h [24]. The final mixture was heated by the LIRS method (approximately 90 °C). The resulting rGO-containing MOF composite crystals were washed, filtered, and dried under a vacuum at 60 °C. Finally, the MOF/rGO composites were obtained (Figure 3).

### 3.3. Electrochemical Performance and Electrical Conductivity Characterization

#### Electrochemical Analysis of NiCo (2:1–1:2) Bimetallic MOFs

The electrochemical measurements were performed at room temperature using the Palmsens EmStat-4 C-ES4S-LR.F0 potentiostat/galvanostat. Electrochemical studies on the prepared MOFs have been performed using cyclic voltammetry (CV), galvanostatic charge–discharge (GCD), and electrochemical impedance spectroscopy (EIS) tests using a three-electrode system in 0.5 M HCl and 0.1 KOH electrolytes. In this three-electrode system, a glassy carbon electrode (GCE, d: 3 mm) acts as the working electrode, silver/silver chloride (Ag/AgCl) as the reference electrode, and a Pt (platinum) wire as the counter electrode. A 2% wt. Nafion solution acted as a binder and ethanol/DMF (3:1) as a solvent. First, the CV test was performed at various scan rates of 10–500 mV s^−1^ with the GCE, without applying any catalytic materials (MOFs). Then, the system was covered with catalytic materials (2 mg/200 mL) and left at room temperature. EIS tests were conducted under potentiostatic conditions using the three-electrode system. This is an important technique that measures the behavior of electrochemical systems as electrical resistors and capacitors through the frequency domain. The measurements started from a high-frequency range (100 kHz) down to a low-frequency range (0.01 Hz) with an AC voltage of 5.0 mV. All these analyses were carried out on Ni2Co1MOF, Ni1Co2-MOF, rGO-Ni2Co1MOF, and rGO-Ni1Co2MOF samples having rGO amounts of different proportions.

The specific capacitances (Cs, Fg^−1^) of the electrodes were calculated from CV tests according to Equation (1) below:

(1) $$C_{s,\;CV}=\frac{\int I.dV}{m.\nu .\Delta V}$$

The specific capacitance (C_s_) was calculated from the GCD curves using Equation (2). In this equation, *I* is the discharge current (A), *Δt* is the discharge time (s), *m* is the mass of the active material (g), and *ΔV* is the potential window (*V*).

(2) $$C_{sp}=\frac{I.\Delta t}{m.\Delta V}$$

### 3.4. Electrochemical Analysis of Powdered MOF, MOF/rGO Composites, and Acrylate/MOF Composites Prepared by In Situ Photopolymerization Method

#### 3.4.1. Dielectric Measurements of Ni-Co MOF (2:1–1:2) and MOF/rGO (1–10%)

Powdered Ni-Co MOF (2:1–1:2) and MOF/rGO (1–10%) composites were coated onto the ITO glass surface used as a substrate via the drop method and dried at 60 °C (Figure S1), and a parallel-plate capacitor geometry was used to measure their dielectric properties. A Ag film was coated onto the prepared coatings as the top plate under pressure of 10^−5^ Torr, and a sandwich geometry was created. Dielectric measurements were performed at room temperature in the frequency range of 10 to 10^6^ Hz.

#### 3.4.2. Dielectric Characterization of UV-Cured PEGMEA/PEGDA/MOF and PEGMEA/PEGDA/rGO–MOF Thin Films

**Figure 4:**
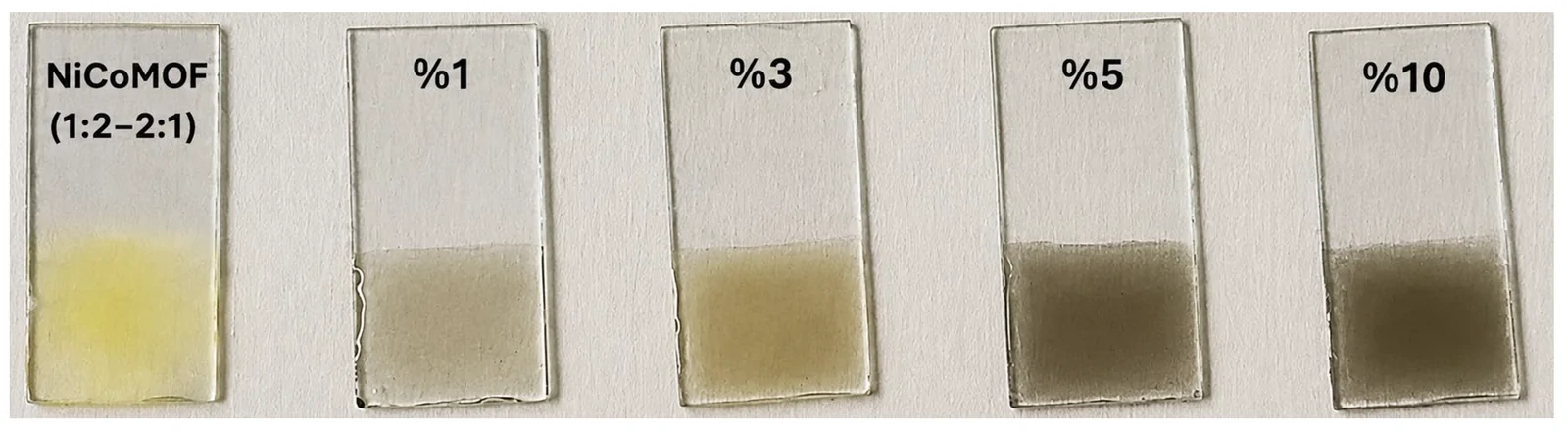
UV-cured PEGMEA/PEGDA/NiCo-MOF (Ni/Co = 2:1–1:2) and PEGMEA/PEGDA (1–10 wt.%) rGO–NiCoMOF (Ni/Co = 2:1–1:2) coatings on ITO substrates.

The thermal and morphological characterization of the photochemically prepared polymeric films (Figure 4) was carried out using a monomer mixture consisting of PEGMEA (30 wt.%; Aldrich, *M*_n_ = 550) and PEGDA (70 wt.%; Aldrich, *M*_n_ = 700). The formulation contained the one-component type II photoinitiator TXSCH_2_COOH (0.02 wt.%) and was prepared in both the absence and the presence (0.01 wt.%) of Ni-Co MOFs with Ni/Co ratios ranging from 1:2 to 2:1.

The thermal and morphological characterization of photochemically prepared polymeric films (Figure 4) was performed using a monomer mixture of PEGMEA (30 wt.%, Aldrich, Mn = 550) and PEGDA (70 wt.%, Aldrich, Mn = 700), with a one-component type II photoinitiator (TXSCH_2_COOH) (0.02 wt.%) and Ni-Co MOFs (1:2–2:1) both present (0.01 wt.%) and absent. In addition, polymeric nanocomposites of the aforementioned MOFs with rGO were prepared using the PEGMEA/PEGDA monomer mixture.

To achieve the homogeneous dispersion of the MOFs in the PEGMEA/PEGDA monomer mixture, the formulation was treated using both an ultrasonic probe and an ultrasonic bath. Subsequently, 100 μL of the suspension was deposited onto an ITO-coated glass substrate by spin coating and photocured using a mini-UV curing system to obtain thin polymeric films (Figure S2).

A parallel-plate capacitor configuration was employed to measure the dielectric properties of the prepared thin polymeric films. Polymer-only, MOF/polymer, and rGO-MOF/polymer composite films were deposited onto ITO-coated glass substrates by spin coating. Subsequently, a silver (Ag) top electrode was deposited onto the polymer films under a vacuum of 10^−5^ Torr, resulting in a sandwich-structured capacitor. Dielectric measurements were carried out at room temperature over the frequency range of 10 to 10^7^ Hz.

## 4. Results and Discussion

### 4.1. Characterization and Investigation of the Photophysical and Photochemical Properties of Bimetallic Ni-Co MOFs (Ni/Co = 2:1–1:2)

#### 4.1.1. FTIR Spectroscopy and XRD Analysis

**Figure 5:**
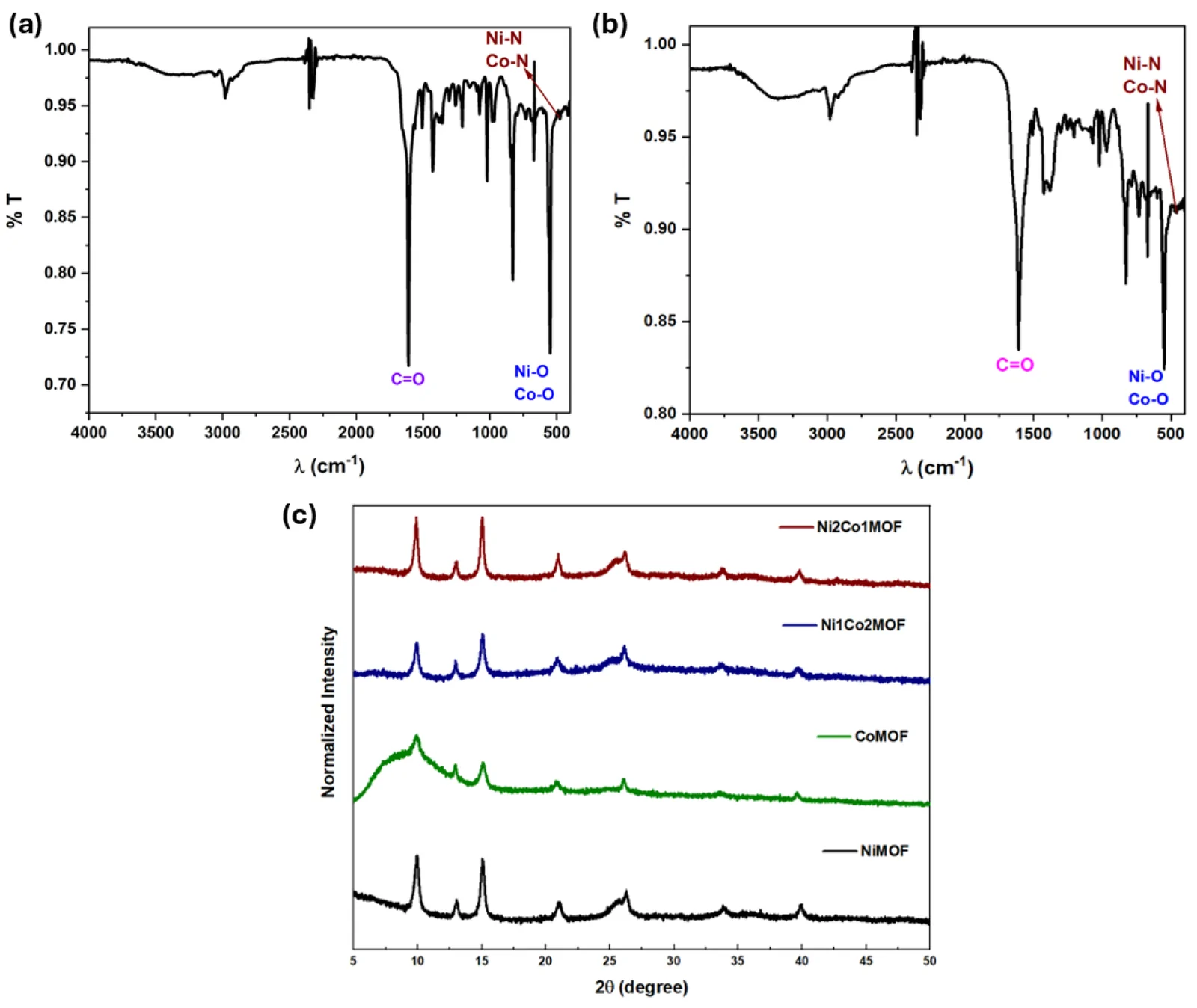
(**a**) FTIR spectra of Ni2Co1MOF and (**b**) Ni1Co2MOF. (**c**) XRD spectra of NiMOF, CoMOF, Ni1Co2MOF, and Ni2Co1MOF structures.

The FTIR spectroscopy analysis of Ni2Co1MOF and Ni1Co2MOF is given in Figure 5a,b. Intense bands between 1600 cm^−1^ and 1420 cm^−1^ correspond to asymmetric and symmetric stretching vibrations of the carboxylate ions (COO–) of H_2_L. The lack of C=O in carboxylic acid at ~1710–1730 cm^−1^ suggests that the linkers are deprotonated and coordinated [25].

C-O stretching vibration is characterized by a band at 1022 cm^−1^, while the moderately intense one at 828 cm^−1^ corresponds to the aromatic C-H out-of-plane bend. The band at 546 cm^−1^ corresponds to the Ni-O/Co-O bonds of the carboxylate ions. The moderate band at 478 cm^−1^ represents Ni-N/Co-N bond formation, where the coordination takes place via N donors. Hence, the FTIR spectroscopy analysis confirms the M-O and M-N coordination in the Ni/Co metal organic frameworks, with no free acid terminations in the sample. The Δν value of approximately 180 cm^−1^ indicates the bridging coordination of the carboxylate groups to the metal centers, confirming the formation of a bridging carboxylate-linked Ni-Co MOF [26]. The small fluctuations observed around 2300–2400 cm^−1^ are attributed to residual atmospheric CO_2_ and do not interfere with the characteristic peaks of the samples.

The powder XRD plots of NiMOF, CoMOF, Ni1Co2MOF, and Ni2Co1MOF for the angle range of 2θ = 5–50° are presented in Figure 5c. The crystalline phase of the materials is clearly observed. Reflections are observed at the low angle range of 2θ = 7–10° and reflections are also present at angles of 2θ = 21–26° and 40–42° in each plot; therefore, based on the XRD plots, the samples contain similar peak locations [27]. The levels of crystallinity for all structures can be ordered as NiMOF ≳ Ni2Co1MOF > Ni1Co2MOF > Co-MOF, based on the density/sharpness of the peaks. Our work on NiMOF [28] indicated that the MOF possessed a primitive triclinic structure with P1 space groups without central symmetry.

Despite the relatively low crystallinity of CoMOF, its characteristic diffraction peaks remain at nearly identical 2θ positions (approximately 10°, 13°, 15°, 21°, and 26°) to those observed for NiMOF and the bimetallic MOFs, confirming the preservation of the same crystal framework topology. The broad amorphous halo centered at approximately 2θ ≈ 9° suggests the presence of partially disordered regions or coordination defects within the CoMOF framework. This behavior is likely associated with the different coordination kinetics and nucleation behavior of Co^2+^ ions compared with Ni^2+^ during the laser-induced rapid synthesis process, which may limit the development of long-range crystallographic order. In contrast, the incorporation of Ni^2+^ into the framework significantly suppresses the amorphous contribution, leading to sharper diffraction peaks and improved structural ordering in the bimetallic MOFs. These observations suggest a synergistic interaction between Ni^2+^ and Co^2+^ ions that promotes crystal growth while preserving the original MOF framework topology.

#### 4.1.2. XPS Analysis

**Figure 6:**
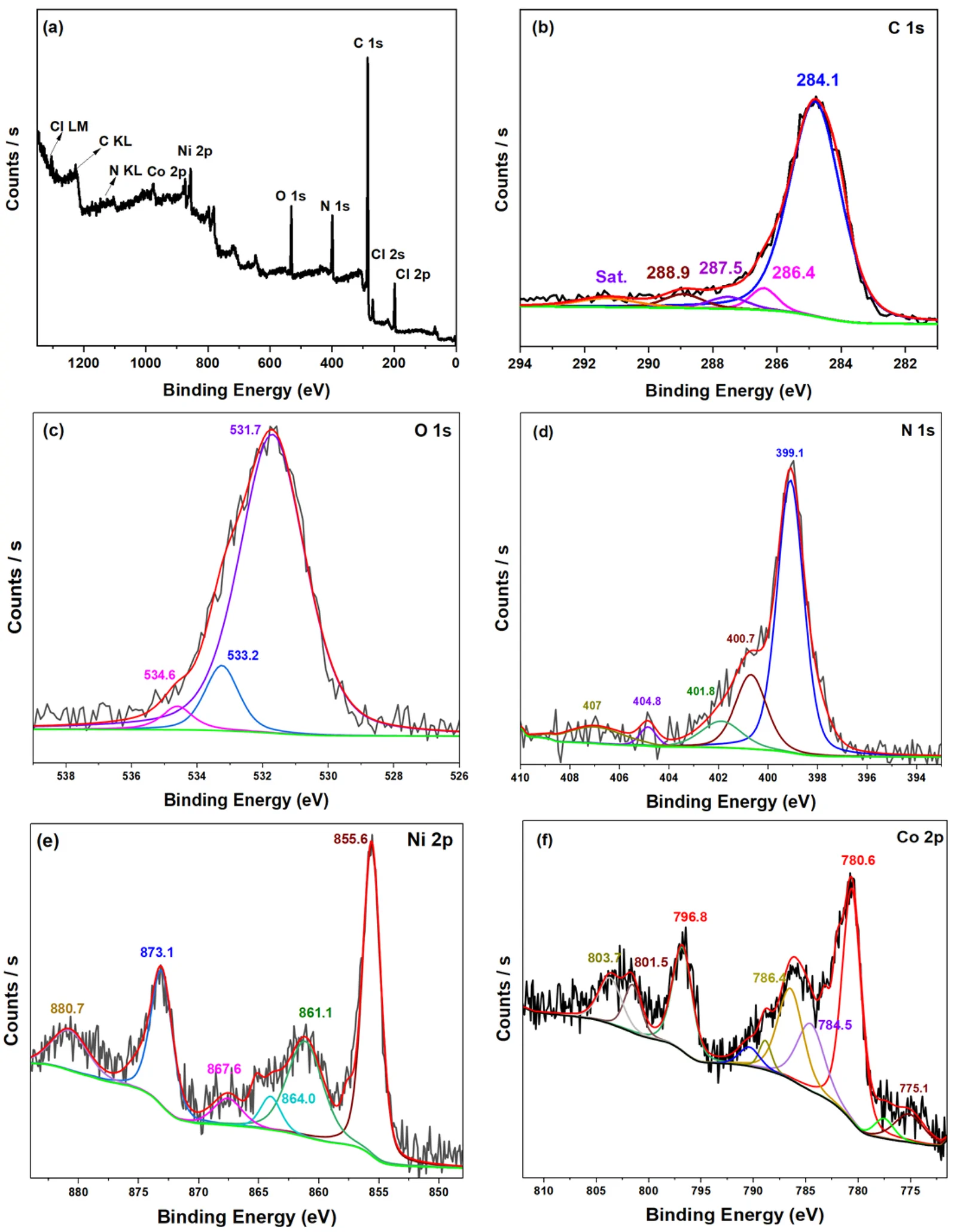
XPS graphs of Ni2Co1MOF: (**a**) general scan spectrum of Ni2Co1MOF and (**b**) C1s, (**c**) O1s, (**d**) N1s, (**e**) Ni2p, and (**f**) Co2p binding energies.

The XPS spectra of Ni2Co1MOF are presented in Figure 6a–f. The C 1s spectrum (Figure 6b) exhibits a dominant peak at 284.1 eV, corresponding to C–C/C=C bonds of the aromatic linker [29]. The peak at 286.4 eV is assigned to C–N/C–O bonds [12,30,31], while the component at 287.5 eV corresponds to C=O or N–C=O species [29,32]. The peak at 288.9 eV is attributed to the O–C=O group of coordinated carboxylates, confirming the coordination between the organic linker and the metal ions [33]. A weak satellite feature observed at 291–292 eV originates from the π–π* transition of the conjugated aromatic carbon, confirming the preservation of the π-conjugated ligand structure. The O 1s spectrum (Figure 6c) consists of three components. The dominant peak at 531.7 eV corresponds to lattice oxygen coordinated to Ni and Co (Ni–O/Co–O) within the framework [34,35]. The peak centered at 533.2 eV is assigned to C–O bonds [36], whereas the weak component at 534.6 eV originates from physically adsorbed water molecules [37]. The N 1s spectrum (Figure 6d) shows two characteristic components. The peak at 399.1 eV is attributed to nitrogen atoms coordinated to the metal centers [38,39] whereas the component at 400.7 eV corresponds to protonated or metal-activated nitrogen. The shift in the nitrogen binding energy toward 400–401 eV confirms the successful coordination of the nitrogen-containing ligand within the MOF framework.

The high-resolution Ni 2p spectrum (Figure 6e) was deconvoluted into its individual components. The dominant Ni 2p_3/2_ peak located at 855.6 eV is characteristic of Ni^2+^ coordinated within the MOF framework. Notably, no peak or shoulder is observed near the characteristic binding energy of metallic Ni^0^ (~852.6 eV), excluding the presence of zero-valent nickel. This indicates that nickel remains coordinated to the organic linker rather than forming metallic nanoparticles during the laser-induced rapid synthesis process. Similarly, the Co 2p spectrum (Figure 6f) was deconvoluted into individual components. The main Co 2p_3/2_ peak appears at 780.6 eV, confirming the predominance of high-spin Co^2+^ species coordinated to oxygen and nitrogen atoms in the framework. Although a very weak feature is observed at approximately 775.1 eV, its intensity is close to the background noise level and it does not correspond to metallic Co, which is typically detected near 778.0 eV. Instead, this feature is attributed to minor surface reconstruction, defect sites, or the instrumental background rather than metallic cobalt. Furthermore, the absence of metallic Ni and Co diffraction peaks in the XRD patterns, together with the TEM results, confirms that the framework retains its bimetallic MOF coordination structure after laser synthesis.

**Figure 7:**
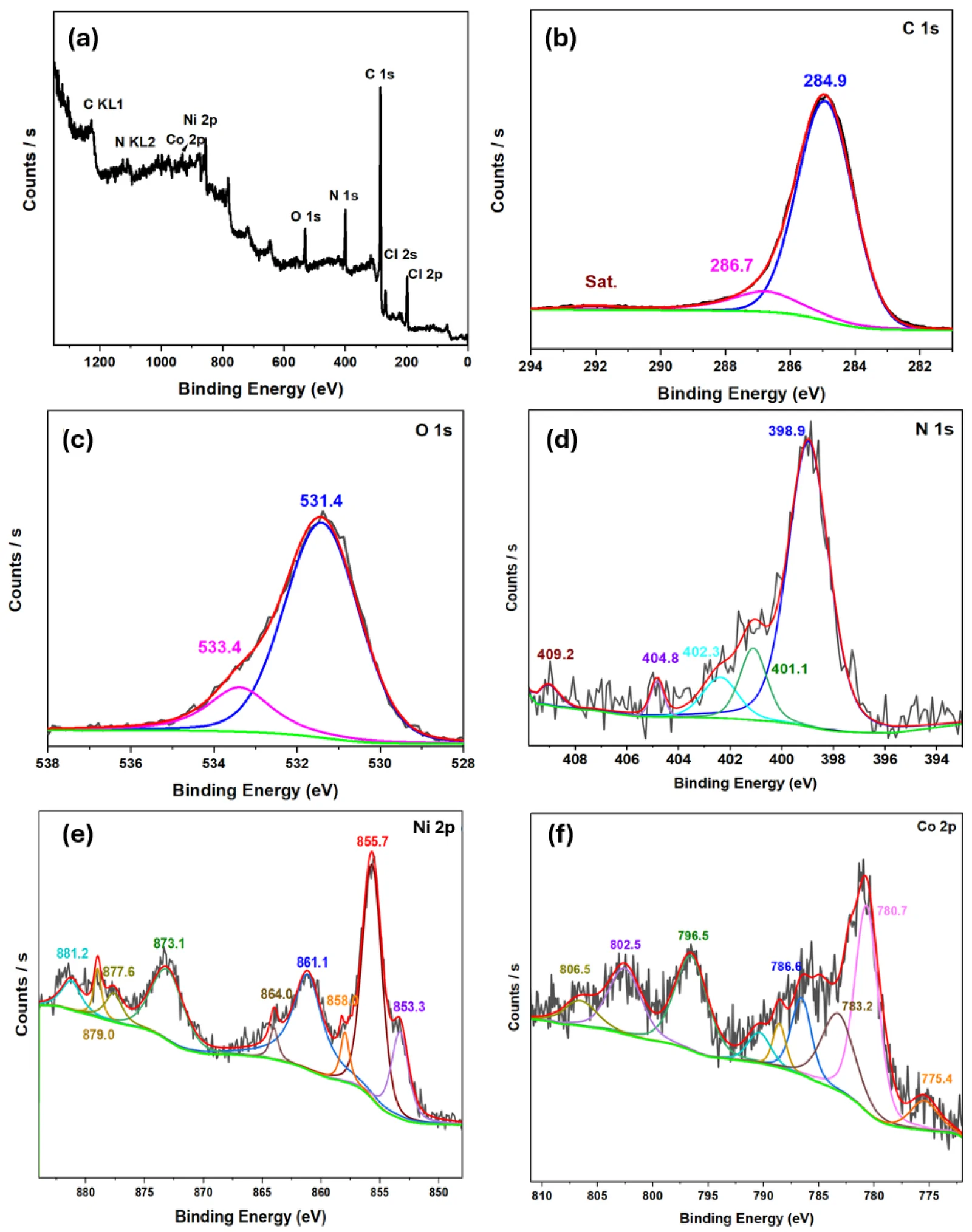
XPS graphs of Ni1Co2MOF: (**a**) overall scan spectrum of Ni1Co2MOF and (**b**) C1s, (**c**) O1s, (**d**) N1s, (**e**) Ni2p, and (**f**) Co2p binding energies.

The XPS spectra of Ni1Co2MOF are presented in Figure 7a–f. The C 1s spectrum (Figure 7b) shows a dominant peak at 284.9 eV assigned to C–C/C=C (sp^2^/sp^3^) bonds originating from the aromatic organic linker [29]. The component centered at 286.7 eV corresponds to C–N and C–O bonds of the coordinated ligand [29,32]. A weak satellite peak at 290–292 eV is attributed to the π–π* transition of conjugated aromatic carbon. The O 1s spectrum (Figure 7c) consists mainly of the Ni–O/Co–O component centered at 531.4 eV [34,35], while the peak at 533.4 eV is assigned to C–O bonds of the ligand [36]. The N 1s spectrum (Figure 7d) exhibits a peak at 398.9 eV corresponding to nitrogen atoms coordinated to the metal centers, which is consistent with nitrogen-containing MOFs. The component at 401.1 eV is assigned to protonated or metal-coordinated nitrogen [38,40]. Weak components observed at 404.8 and 409.2 eV are attributed to trace N–O species.

The deconvoluted Ni 2p spectrum (Figure 7e) confirms that Ni Is predominantly present in the Ni^2+^ oxidation state. The principal Ni 2p_3/2_ peak at 855.7 eV as well as its satellite features at 861.1 eV and the Ni 2p_1/2_ component at 873.1 eV are characteristic of Ni^2+^ in a ligand-coordinated environment. An additional deconvoluted component appears at 853.3 eV. Although this peak occurs at a lower binding energy than the main Ni^2+^ peak, it is still significantly higher than the characteristic binding energy of metallic Ni^0^ (~852.6 eV). Therefore, this feature is assigned to low-coordination surface Ni sites or strong metal–ligand electronic interactions within the MOF framework, rather than metallic nickel clusters formed during laser synthesis.

The deconvoluted Co 2p spectrum (Figure 7f) is dominated by the Co 2p_3/2_ peak at 780.7 eV, characteristic of high-spin Co^2+^ coordinated within the framework. A very weak feature appears at approximately 775.4 eV; however, its intensity is close to the background noise and it does not coincide with the characteristic metallic Co binding energy (~778.0 eV). Accordingly, this weak contribution is attributed to minor surface defects or the instrumental background instead of metallic cobalt.

The high-resolution XPS spectra confirm that the predominant oxidation states of the metal centers are Ni^2+^ and Co^2+^. The deconvoluted Ni 2p and Co 2p spectra, together with their characteristic satellite peaks, are consistent with divalent Ni and Co coordinated by O- and N-donor ligands. Although weak low-binding-energy features are observed, they do not coincide with the characteristic binding energies of metallic Ni^0^ (~852.6 eV) or metallic Co^0^ (~778.0 eV). Instead, these minor components are attributed to low-coordination surface sites, defect-related species, or background contributions rather than zero-valent metals. Furthermore, the absence of metallic diffraction peaks in the XRD patterns supports the conclusion that no metallic Ni or Co phases were formed during the laser-induced synthesis.

### 4.2. Morphological Characterization

**Figure 8:**
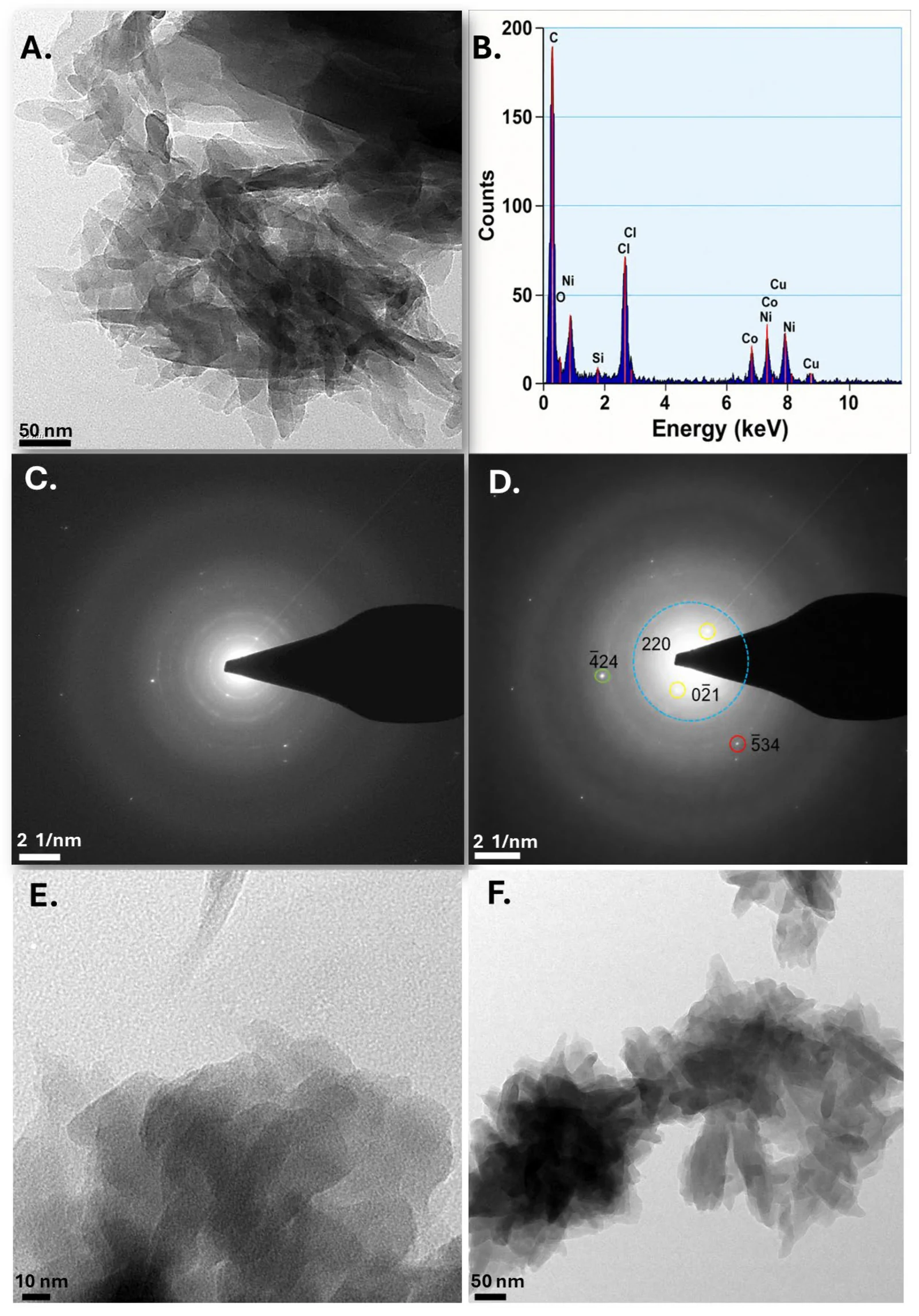
Transmission electron microscopy (TEM) characterization of the bimetallic MOFs: (**A**) TEM image of Ni2Co1MOF, (**B**) energy-dispersive X-ray spectroscopy (EDS) spectrum of Ni2Co1MOF, (**C**) selected area electron diffraction (SAED) pattern of Ni2Co1MOF, (**D**) indexed crystal planes corresponding to the SAED pattern of Ni2Co1MOF, and (**E**,**F**) TEM images of Ni1Co2MOF.

As a secondary method for controlling the crystal structure, the selective field electron diffraction (SAED) technique integrated with transmission electron microscopy (TEM) was used [41,42,43]. The advantage of this technique over XRD is that it allows the material to be examined morphologically with TEM, elementally with energy-dispersive X-ray spectrometry, and structurally with SAED simultaneously and locally. As an example, Figure 8 presents the TEM image, EDS spectrum, raw SAED image, and hkl values in the planes corresponding to the relevant scatterings of a Ni2Co1 MOF sample. The lattice spacings of these planes are approximately 5.41 Å, 3.34 Å, 2.06 Å, and 1.69 Å, respectively, and the hkls are obtained as 0–21, 220, −424, and −534. In conclusion, these lattice constants are simultaneously present in the primitive triclinic structure with the P1 space group and lacking central symmetry, as mentioned in our previous studies [28]. PXRD analysis was performed by evaluating the TEM results together with morphological and crystalline properties. The EDS image of Ni2Co1MOF in Figure 8B shows presence of Co and Ni confirming relatively larger amount of Ni.

**Figure 9:**
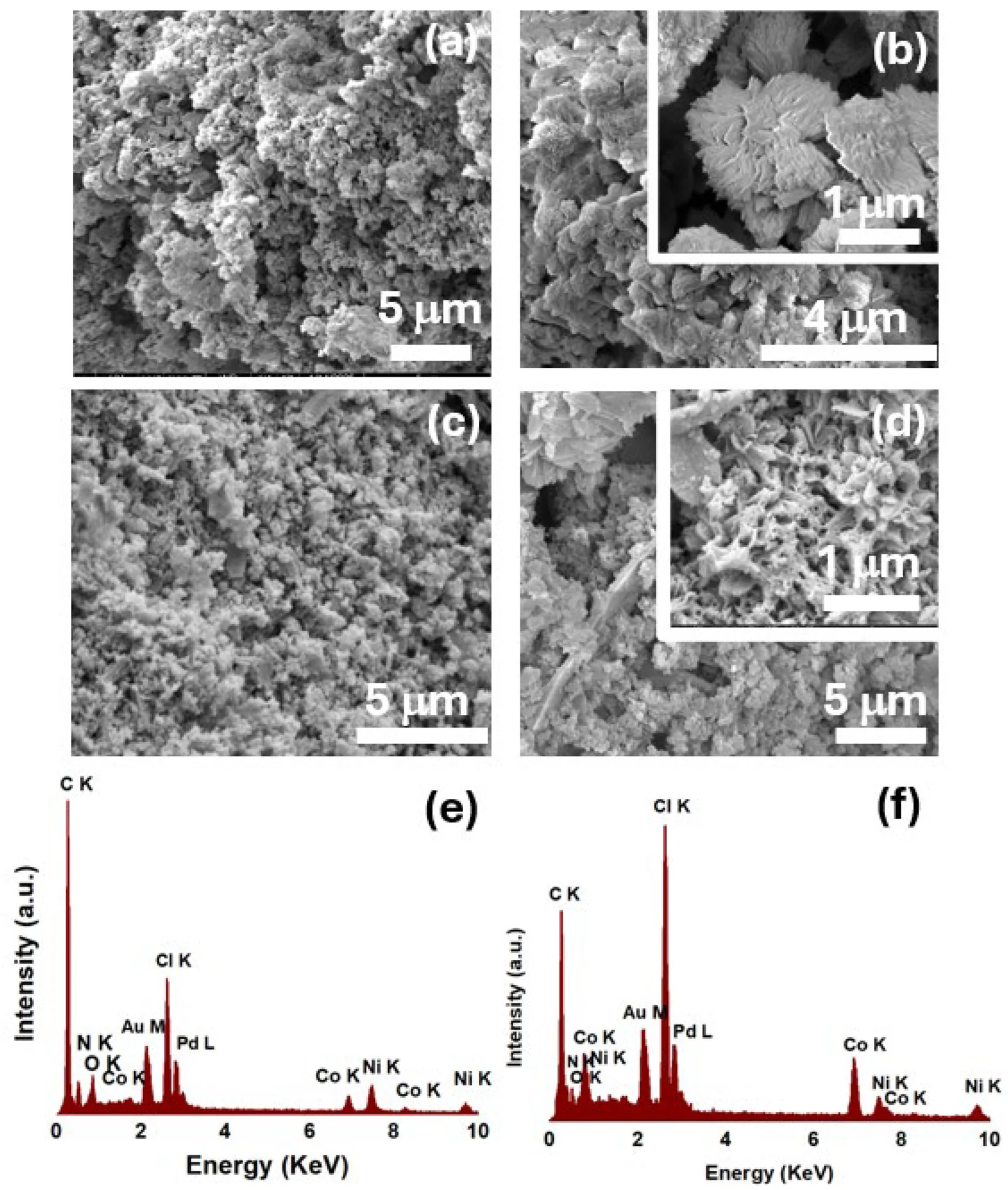
SEM micrographs of Ni2Co1MOF at different magnifications (**a**,**b**) and Ni1Co2MOF at different magnifications (**c**,**d**), together with the corresponding EDX spectra of Ni2Co1MOF and Ni1Co2MOF (**e**,**f**), confirming the elemental compositions of the synthesized bimetallic MOFs.

The SEM images (Figure 9a,b) reveal that Ni2Co1MOF exhibits a hierarchical morphology composed of cauliflower-like aggregates together with flower-like sheet structures formed by intergrown nanosheets. Similarly, Ni1Co2MOF displays highly branched cauliflower-like architectures consisting of interconnected subunits (Figure 9c,d), consistent with previous reports [8]. The rough and fragmented surface morphology of both MOFs is attributed to rapid nucleation followed by the aggregation of primary particles through a cluster–cluster growth mechanism, resulting in the formation of larger bimetallic MOF aggregates. The EDS spectra of Ni2Co1MOF and Ni1Co2MOF (Figure 9e,f) confirm the presence of Ni and Co as the principal metallic constituents and verify the expected Ni/Co atomic ratios. The pores of Ni2Co1MOF were determined to be more numerous and larger compared to the other case.

### 4.3. Optical Properties

**Figure 10:**
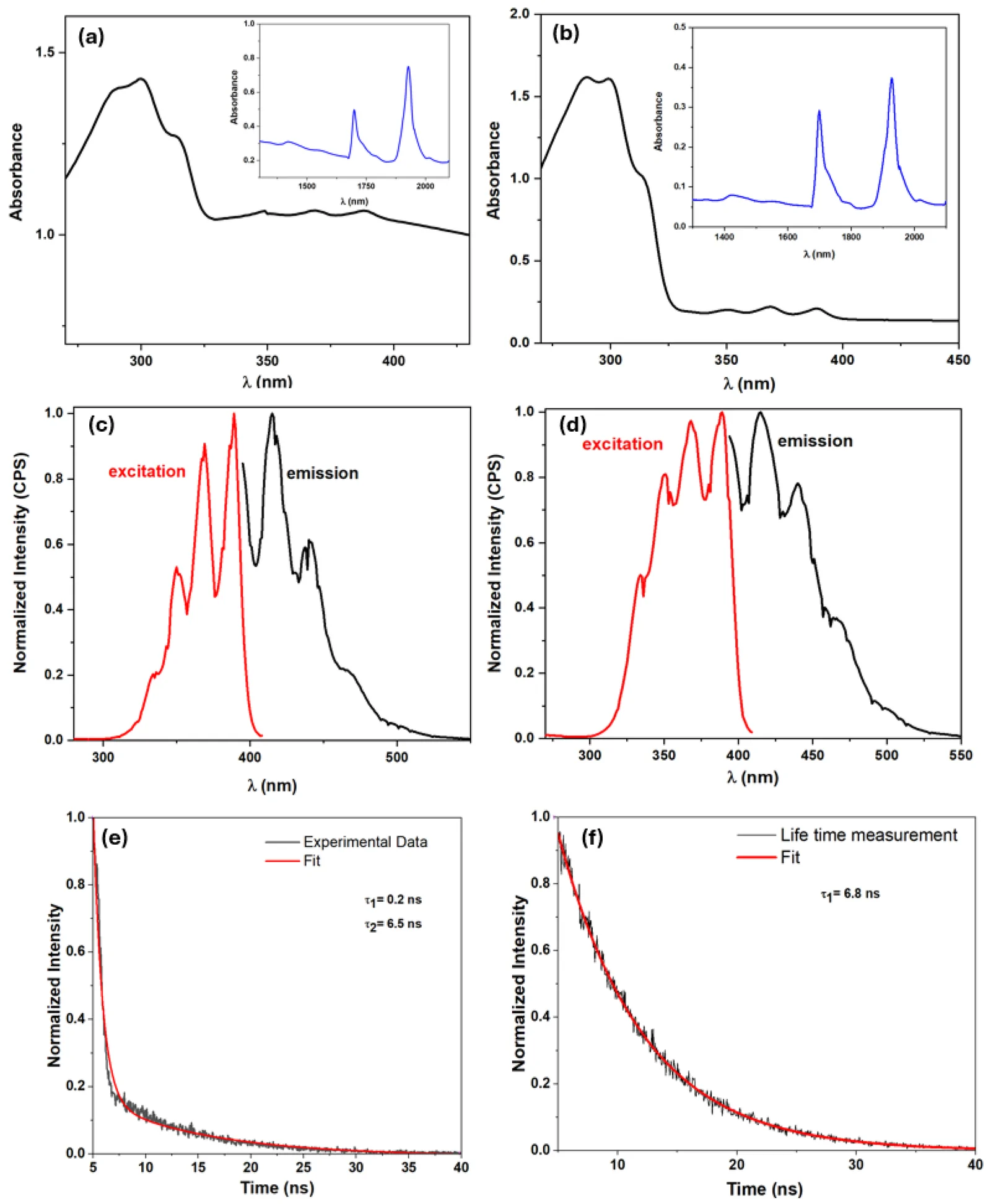
UV-VIS-NIR absorption spectra of Ni2Co1 and Ni1Co2MOFs in DMF (1 mg/3 mL): (**a**,**b**) absorption spectra; (**c**,**d**) fluorescence excitation (λ_abs_ = 389 nm) and emission (λ_exc_ = 415 nm) spectra recorded at room temperature; and (**e**,**f**) time-resolved fluorescence decay curves. The insets in (**a**,**b**) are the NIR absorption of the Ni2Co1 and Ni1Co2MOFs.

In the UV-NIR spectrum of Ni2Co1MOF in Figure 10a, there is a noticeable UV band (270–330 nm) whose peaks occur at 290 nm, 299 nm, and 314 nm due to the ligand π→π* transition from the aromatic bpe ligand to the metals. The slight break point in the peaks occurs at 290, 299, and 314 nm, revealing the existence of several ligands in the frame. The weak bands in the NIR (1422 nm, 1699 nm, and 1928 nm) indicate the d–d transitions of high-spin Ni^2+^ and Co^2+^ ions. This happens because these bands represent d–d transitions taking place in octahedrally distorted regions.

The region from 288 to 389 nm In UV shows the π–π* and n–π* transitions of bpe and H_2_L. On the other hand, the NIR (1420–1929 nm) depicts metal-centered d–d transitions (Ni^2+^/Co^2+^), which occur due to charge transfer effects. Therefore, the spectrum indicates the successful incorporation of Ni and Co into the MOF, leading to an increase in absorption, which may be beneficial for photocatalytic or photothermal processes (Figure 10b). In particular, there are the π–π* transitions of bpe at 288 nm and 299 nm.

From the absorption from aromatic π orbitals to π*, one can conclude that there are strong coordinates of metal centers in bpe and conjugations as well. n–π* or metal–ligand charge transfer (MLCT) transitions at 350 nm, 368 nm, and 389 nm occur when there is coordination of H_2_L to Ni^2+^ and Co^2+^ ions. The d–d transitions and M–M charge transfers (NIR) at 1420 nm, 1700 nm, and 1929 nm happen due to Ni^2+^ (d^8^)/Co^2+^ (d^7^) from the bridge of Ni–O–Co through the linker. In general, MOFs incorporating Ni and Co or their bimetallic variants have similar UV-VIS-NIR properties.

The fluorescence spectrum of Ni2Co1MOF (Figure 10c) has a resemblance to that of a binder-weighted chromophore that emits rigid blue light with slight overlap among excitation–emission. Because of its lower Co than Ni1Co2MOF, it avoids quenching and emits blue light upon being excited by UV rays. The same property is shown by Ni1Co2MOF containing a Co component (Figure 10d). Upon examining the emission time decay curves of Ni2Co1MOF and Ni1Co2MOF (Figure 10e,f) separately, Ni2Co1MOF emits at half-lives of 0.2 ns and 6.5 ns, while the other does so at 6.8 ns.

The fluorescence decay curves shown in Figure 10f were analyzed using time-correlated single-photon counting (TCSPC). The experimentally observed fluorescence signal was analyzed after convolution with the instrument response function (IRF) according to

(3) $$I_{obs}\left(t\right)=IRF\left(t\right).l(t)$$

where $I_{obs}(t)$ is the measured fluorescence intensity, IRF(t) is the instrument response function, and ⊗ denotes the convolution operation [44,45]. Depending on the decay characteristics, the fluorescence lifetime data were fitted using either single- or bi-exponential decay models [44].

The fluorescence decay profile of Ni2Co1MOF was best described by a bi-exponential decay model

(4) $$I(t)=A_{1}e^{-t/\tau_{1}}+A_{2}e^{-t/\tau_{2}}+C$$

where $A_{1}$ and $A_{2}$ are the amplitudes of the two decay components, $\tau_{1}$ and $\tau_{2}$ are the corresponding fluorescence lifetimes, and C is the background constant. The fitting yielded lifetime components of τ_1_ = 0.190 ns and τ_2_ = 6.553 ns, with relative amplitudes of 52.19% and 47.81%, respectively. The reduced chi-square value (χ^2^ = 0.916) confirms the excellent agreement between the experimental data and the fitted curve. The presence of two lifetime components indicates two distinct emissive environments or recombination pathways within the Ni2Co1MOF framework.

In contrast, the fluorescence decay of Ni1Co2MOF was adequately fitted using a single-exponential decay model

(5) $$I(t)=Ae^{-t/\tau }+C$$

where A is the decay amplitude, τ is the fluorescence lifetime, and C is the background constant. The fitting yielded a fluorescence lifetime of 6.873 ns with a reduced chi-square value of χ^2^ = 0.973, demonstrating an excellent fit between the experimental and calculated decay profiles. The single-exponential decay behavior suggests that fluorescence relaxation in Ni1Co2MOF is dominated by a single emissive species or recombination pathway.

Finally, a comparison of the decay profiles reveals that Ni2Co1MOF exhibits a bi-exponential fluorescence decay, whereas Ni1Co2MOF follows single-exponential decay behavior. This difference indicates that changing the Ni/Co ratio modifies the excited-state relaxation mechanism. The two lifetime components observed for Ni2Co1MOF suggest the coexistence of multiple emissive environments or recombination channels, whereas the single lifetime component of Ni1Co2MOF implies a more homogeneous excited-state relaxation process.

The combined UV-VIS-NIR absorption and fluorescence lifetime results demonstrate that the Ni/Co ratio influences the excited-state relaxation behavior while preserving the characteristic ligand-to-metal electronic interactions within the MOF framework. Such structure–property relationships are consistent with recent studies reporting that the rational modulation of the local electronic environment and interfacial charge transfer processes plays a key role in determining the photophysical and electrical properties of advanced porous materials and MOF-based functional systems. In particular, engineering the electronic structure and charge transfer pathways has been recognized as an effective strategy for improving the optical response, charge separation efficiency, and electrical performance of multifunctional materials, thereby expanding their potential for optoelectronic, photocatalytic, and energy-related applications.

### 4.4. Thermal and N_2_ Adsorption–Desorption Properties

**Figure 11:**
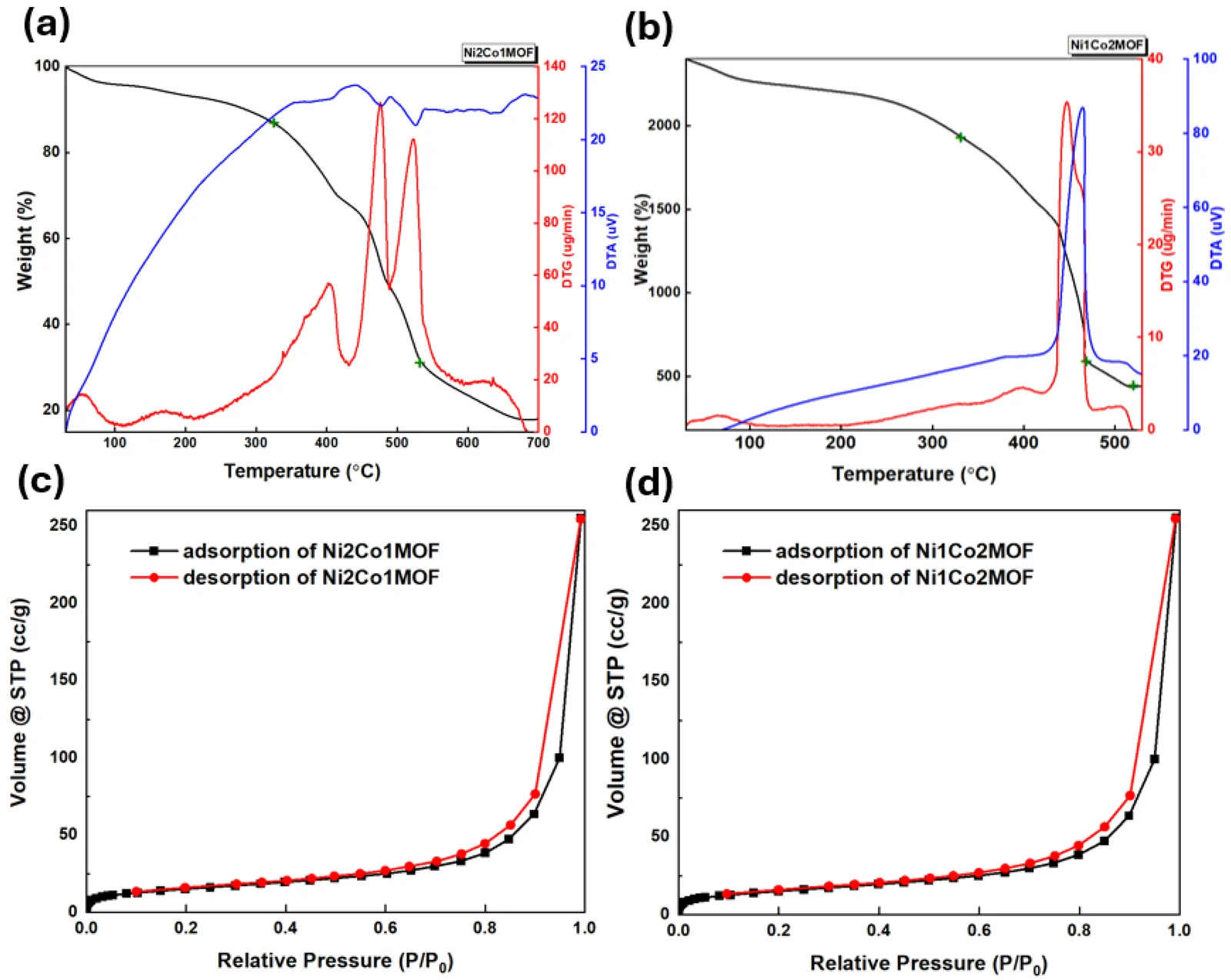
Thermogravimetric (TG) thermograms of (**a**) Ni2Co1MOF and (**b**) Ni1Co2MOF, together with the corresponding N_2_ adsorption–desorption isotherms of (**c**) Ni2Co1MOF and (**d**) Ni1Co2MOF. The black, blue and red lines in (**a**,**b**) stands for the TGA, DTA, and DTG, respectively.

The Ni2Co1MOF thermogram for TGA/DTG/DTA is depicted in Figure 11a. As can be seen, according to the TGA results, a mass loss of 88% and 11.8% at up to 309 °C was detected, whereas, beyond this point, a sharp decrease takes place and results in 29% residue at 542 °C. Specifically, the 11.8% mass loss at 309 °C refers to physical solvent desorption and/or guest detachment. Thus, up to 309 °C, Ni2Co1MOF does not undergo any change. An abrupt mass loss at 420–560 °C can be considered a result of organic linker and Ni/Co node decomposition, which is an exothermic process. At high temperatures above this one, the metal–organic framework transforms into oxides. As the double peak on the DTG graph indicates, there are multiple steps in the MOF degradation process (ligand detachment, carbonaceous material formation, burning). Thus, 560 °C to 600–650 °C is the interval in which the removal of carbonaceous residues takes place and metal oxides are formed; thus, there is almost zero mass loss after this temperature. Figure 11b displays the thermograms for the TGA/DTG/DTA of Ni1Co2-MOF. The residual masses obtained at different temperatures are the following: 95.1–330 °C, 30–469 °C, 20–520 °C, respectively. It should be noted that, according to TGA, there is a total mass loss from approximately 100% of the initial mass to 20% of this at 520 °C. This indicates a 4.9% loss from room temperature to 330 °C due to solvent/moisture detachment. Therefore, the physical desorption of solvents/water and weak guest detachment occurred. Ni1Co2MOF demonstrates stability up to 300 °C without MOF decomposition and even down to 150–180 °C. The MOF mass itself equals 30% at 469 °C.

By analyzing the BET isotherm of Ni2Co1MOF (Figure 11c), the BET specific surface area was determined to be 88.3 m^2^ g^−1^, which is higher than that of Ni1Co2MOF. The total pore volume is 0.022 cm^3^ g^−1^. According to the BJH method, the average pore radius is 16.9 Å (1.69 nm), corresponding to an average pore diameter of 3.38 nm, indicating a small mesoporous structure. The BET specific surface area of Ni1Co2MOF is 52.5 m^2^ g^−1^, with a total pore volume of 0.016 cm^3^ g^−1^. The BJH pore radius is 15.2 Å (1.52 nm), corresponding to an average pore diameter of 3.04 nm. Therefore, Ni1Co2MOF can also be classified as a small mesoporous material. The Ni1Co2MOF exhibits a micro–mesoporous slit-like structure with a relatively low surface area and pore volume (Figure 11d). Compared with Ni1Co2MOF, Ni2Co1MOF exhibits a higher BET surface area (88.3 vs. 52.5 m^2^ g^−1^) and a larger total pore volume (0.022 vs. 0.016 cm^3^ g^−1^). The BJH pore diameters were determined to be 3.38 nm for Ni2Co1MOF and 3.04 nm for Ni1Co2MOF, indicating that both materials are classified as small mesoporous materials (Table 1).

A review of the recent literature related to the synthesis of monometallic Ni-based and Co-based MOFs, as well as bimetallic Ni-Co MOFs, demonstrates that there is an inherent contradiction between stability and porosity in MOF design. Typically, monometallic MOFs, including, for instance, CPO-27-Ni (Ni-MOF-74) and Co-MOF-74-type structures, have relatively high initial microporosity due to the existence of active metal sites; however, they tend to demonstrate poor chemical stability under humid conditions and elevated temperatures [46]. On the other hand, bimetallic Ni-Co MOFs, which are prepared through a conventional one-step solvothermal approach, usually possess lower-than-expected specific surface areas due to the differences in the coordination preferences and crystallization rates of Ni^2+^ and Co^2+^ cations. Such contradictions lead to the formation of heterogeneous nuclei, non-homogeneous crystallization, and partial pore blocking, thus reducing the total porosity of the synthesized materials [47]. Overcoming this issue in the case of bimetallic MOFs requires utilizing the synergistic effects of Ni-Co interaction, achieved through the fine-tuning of the synthesis parameters, such as the metal-to-ligand ratio, modulator addition, and reaction temperature [48].

### 4.5. Preparation of rGO/MOF Composites by In Situ Method

#### 4.5.1. Structural and Morphological Characterization of GO and rGO

In the current study, Ni-Co bimetallic MOFs and their composites with reduced graphene oxide (rGO) were successfully synthesized through an efficient, fast, and continuous-wave LIRS process at temperatures lower than 100 °C. Compared to analogously designed structures synthesized through conventional methods and previously reported in the literature, there are some clear benefits associated with the use of the LIRS process in regard to efficiency, synthesis time, and electrochemical and structural properties.

With respect to synthesis efficiency, it is worth noting that the majority of studies previously conducted have synthesized Ni-Co bimetallic MOFs via conventional solvothermal, hydrothermal, and slow co-precipitation techniques, requiring high temperatures and longer times, from several hours up to 24 h or even longer for successful synthesis. Liquid-based synthesis processes such as solvothermal and hydrothermal reactions involve the use of sealed containers and autogenous pressures, making them energy-intensive and time-consuming. In contrast to these conventional synthesis methods, the current LIRS process provides the rapid synthesis of bimetallic Ni-Co MOFs at lower temperatures than 100 °C and atmospheric pressure.

**Figure 12:**
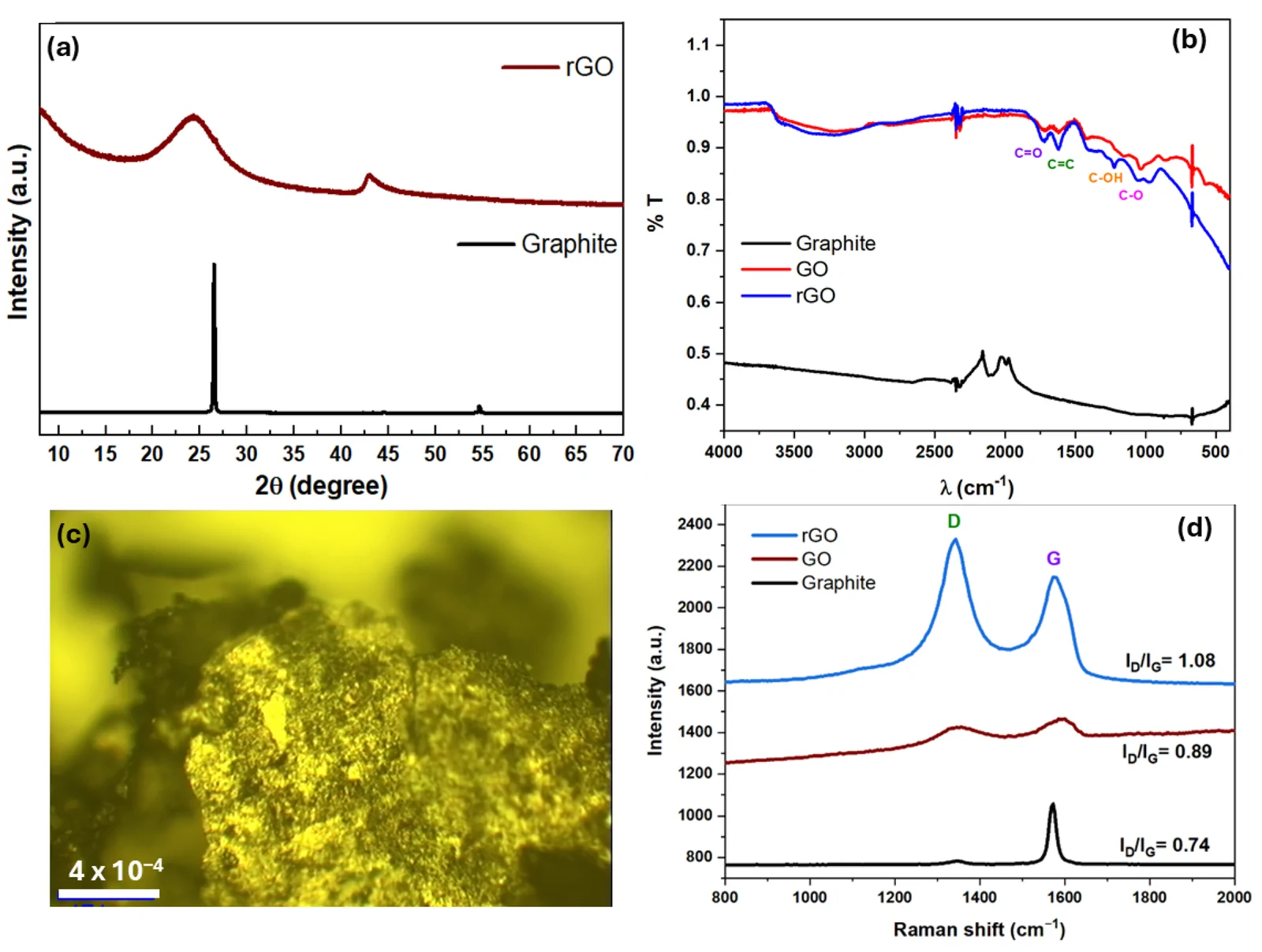
(**a**) Powder X-ray diffraction (PXRD) patterns of graphite and reduced graphene oxide (rGO); (**b**) FTIR spectra of graphite, graphene oxide (GO), and rGO; (**c**) Raman spectra of graphite, GO, and rGO; and (**d**) Raman microscopic image of rGO, confirming the successful oxidation of graphite to GO and its subsequent reduction to rGO. The key peaks of graphene such as D and G peaks are shown in (**d**).

An examination of the XRD results (Figure 12a) shows that they are in complete agreement with the literature [49,50]. When the FTIR spectra are examined (Figure 12b), the formation of -OH peaks is observed in GO at 3000 cm^−1^, while vibrations related to specific reduction are present in rGO between 700 and 2000 cm^−1^. The FTIR results of the structure are in complete agreement with the literature [51,52,53]. The -OH peaks of the FTIR spectra indicate the presence of -OH at 3000 cm^−1^ for GO, whereas specific reduction vibrations can be seen for rGO within the range of 700–2000 cm^−1^.

One of the techniques that is commonly used to characterize carbon-based materials, especially C=C double bonds that cause higher Raman densities, is Raman spectroscopy (Figure 12c,d). Together with electron paramagnetic resonance (EPR) and photoluminescence (PL), Raman spectroscopy can be used as a valuable tool for graphene characterization because of its ability to analyze the structural and electronic properties of graphene, such as the disorder, defect structure, defect density, and addition. The G-band and D-band of GO were determined using Raman spectroscopy and found to appear at 1587 cm^−1^ and 1329 cm^−1^, respectively. Differing from the other applied techniques, the use of Raman spectroscopy provided evidence of structural modifications that occurred both prior to and after the GO reduction using ascorbic acid. As observed, the Raman peaks of rGO exhibited broadening, with the shift of the D- and G-bands to approximately 1342 cm^−1^ and 1577 cm^−1^, respectively. Moreover, the D/G intensity ratio obtained for rGO (1.08) was found to be slightly greater compared to the same ratio for GO (0.97). This means that there was the formation of sp^3^ bonds and an increase in binding defects or the appearance of smaller-sized sp^2^-bound carbon domains [54].

**Figure 13:**
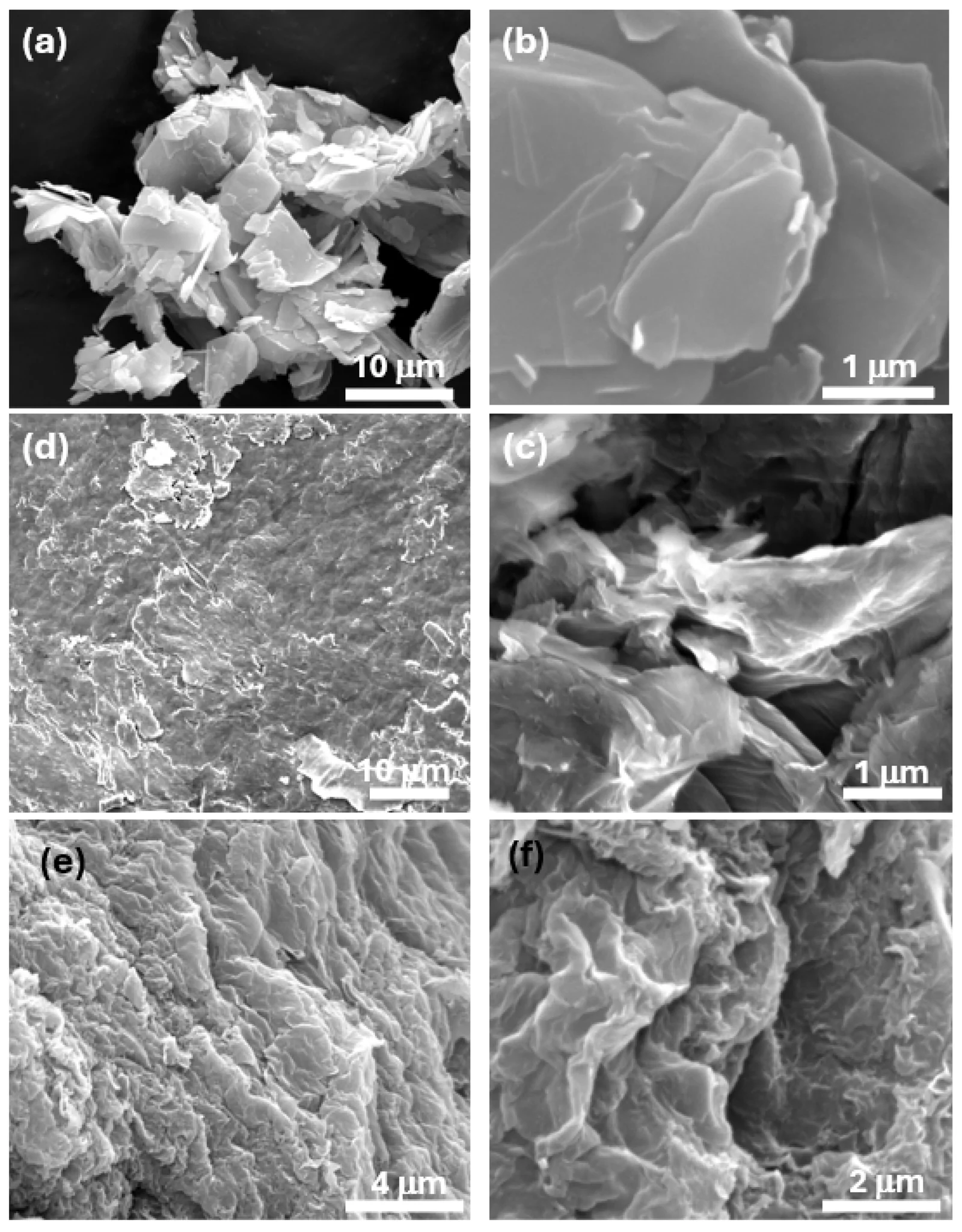
(**a**,**b**) SEM images of graphite, (**c**,**d**) graphene oxide (GO), and (**e**,**f**) rGO.

A comparison of the images obtained using the SEM technique for both GO and rGO is given below. As can be seen from the morphological analysis of the images obtained using the SEM technique, GO possesses a network-like structure formed by interconnected layers of GO nanoplates (Figure 13c,d). According to the SEM images of rGO in Figure 13e,f, the surface of rGO appears to have creases. Such a morphology of the surface of rGO is analogous to a “paper ball-like” structure owing to the isotropic compression and thermal reduction process of the nanomaterial [55].

#### 4.5.2. Structural and Morphological Characterization of rGO/MOF Composites

**Figure 14:**
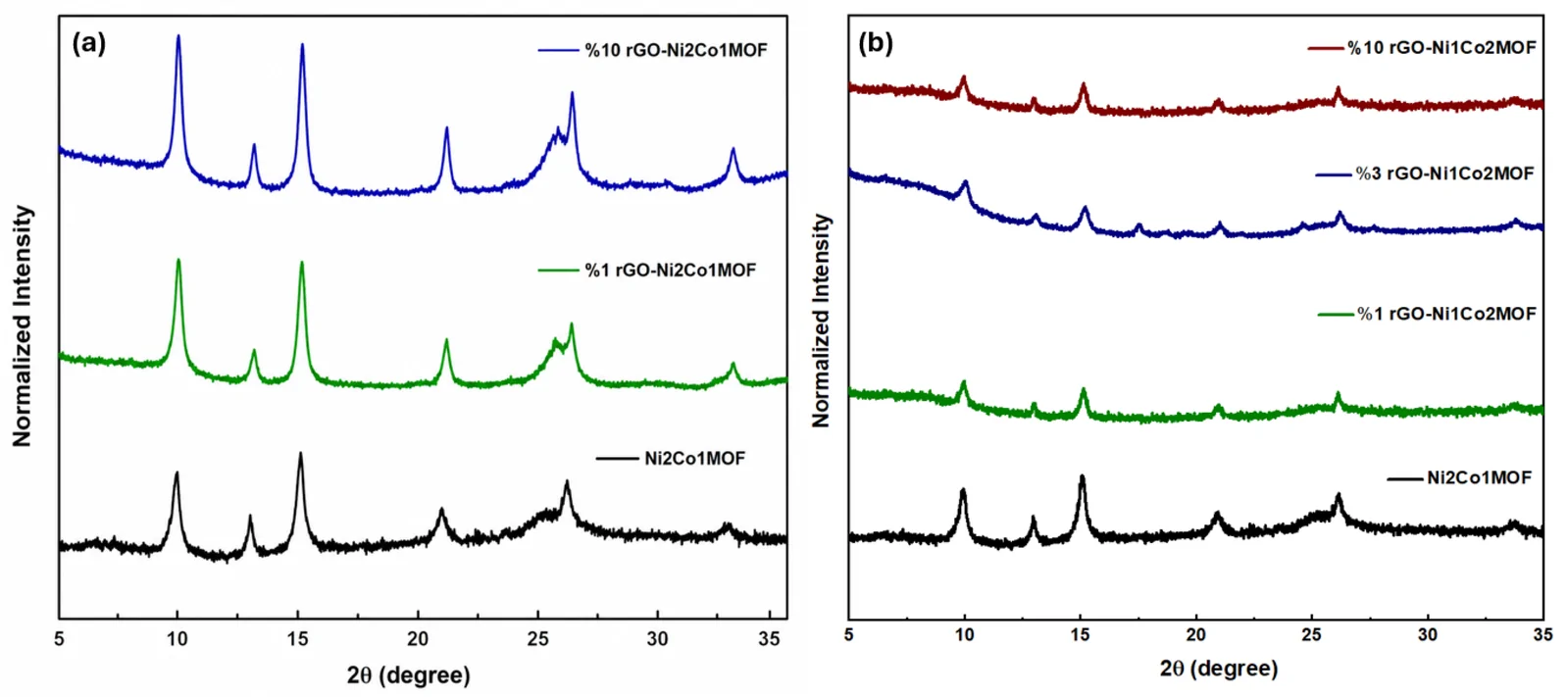
PXRD patterns of (**a**) rGO–Ni2Co1MOF and (**b**) rGO–Ni1Co2MOF composites, illustrating the crystal structure and phase integrity of the MOF framework after the incorporation of rGO.

The XRD spectra of the rGO-Ni2Co1MOF and rGO-Ni1Co2MOF composites are shown in Figure 14. It is observed that the resulting rGO/MOF structures have the characteristic crystal peaks of MOFs.

The MOFs formed using 10% rGO are illustrated in Figure S3. It is evident from the Figure that the composite material in which the boundary of formation resembles a flower ball has a high concentration of nanoflakes owing to the high concentration of rGO used. These nanoflakes can decrease the travel time of electrolyte ions during electrochemical reactions, which creates more active sites for electrochemical processes [8].

### 4.6. Electrochemical Behavior of Bimetallic Ni-Co MOFs in Acidic and Alkaline Media

CV at various scan rates provides information regarding adsorption-/diffusion-based redox mechanisms, which gives vital insights into the redox mechanisms at the MOF-rGO-modified electrode surface in certain ratios (1–10%). EIS was performed at frequencies ranging from 100,000 Hz to 0.1 Hz with the aim of examining the influence of charge transfer resistance at various potentials using Ag/AgCl.

#### 4.6.1. Systematic Electrochemical Analysis of Ni-Co MOFs and rGO–Ni-Co MOF Composites Under Acidic Conditions

##### Electrochemical Characterization of Ni2Co1MOF and rGO–Ni2Co1MOF (1–10 wt.%) Composites by CV, GCD, and EIS

**Figure 15:**
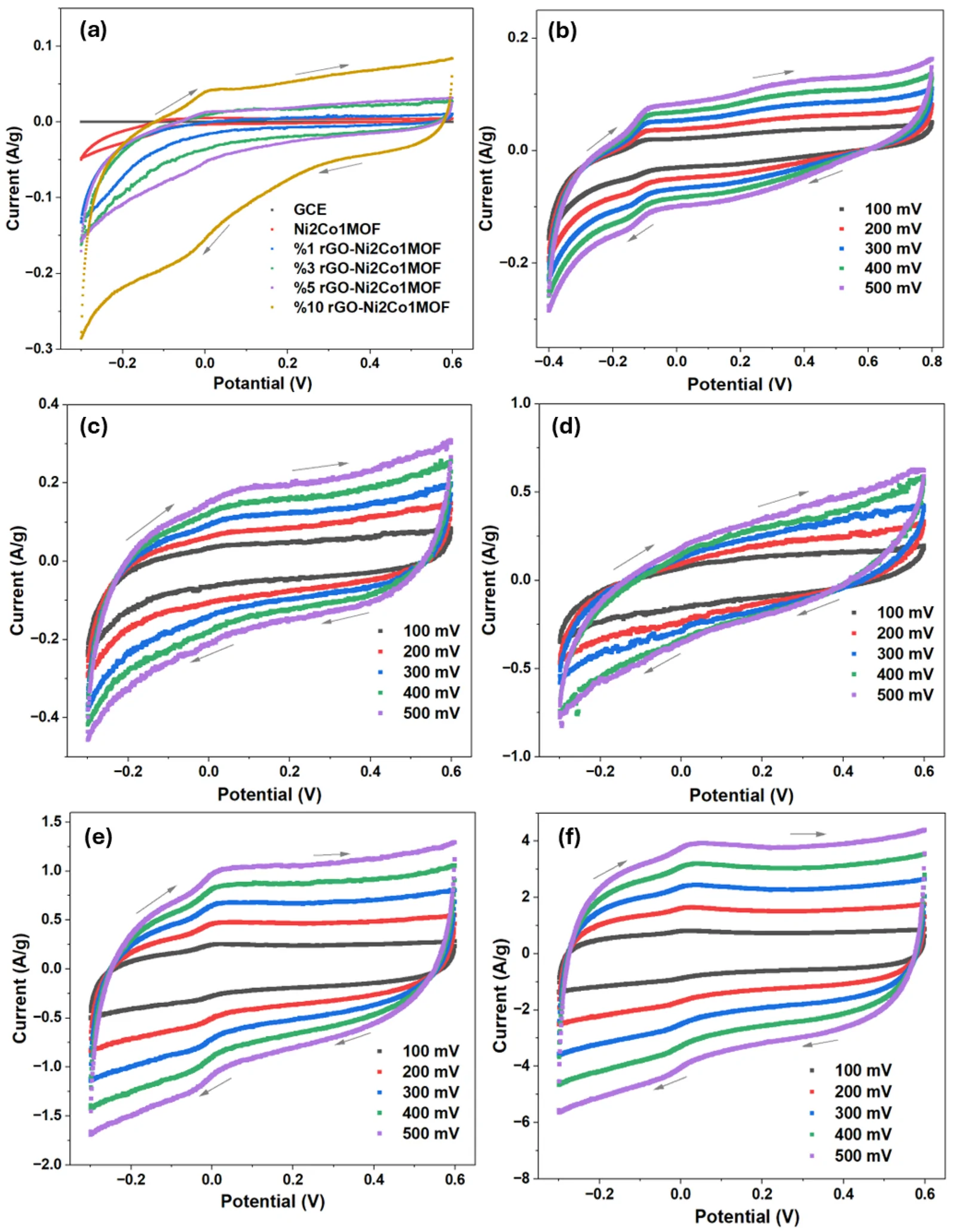
CV curves of GCE electrode, Ni2Co1MOF, and rGO (1–10%)-Ni2Co1MOF composites at 10 mV s^−1^ scan rate (**a**) and at different scan rates (100–500 mV s^−1^) for (**b**) Ni2Co1MOF, (**c**) 1% rGO-Ni2Co1MOF, (**d**) 3% rGO-Ni2Co1MOF, (**e**) 5% rGO-Ni2Co1MOF, and (**f**) 10% rGO-Ni2Co1MOF composites in 0.5 M HCl solution. The arrows show the direction of the current flow in the cyclic voltammetry measurements.

Ni2Co1MOF and rGO-Ni2Co1MOF (1–10%) were studied under acidic conditions in 0.5 M media with different scan rates of 10 and 500 mV s^−1^, respectively, and all curves are plotted in Figure 15. Considering the shapes of the curves in the graphs (Figure 15a–f), one can see that there is strong pseudocapacitive behavior. As the scan rate increased, there was also an increase in current and field broadening depending on the voltage. Thus, this indicates rapid electrochemical responses. The specific capacitance was proportional to the integrated area of the CV curves. In regard to the specific capacitance value at a 10 mV s^−1^ scan rate, the specific capacitance value increased with varying ratios of rGO and Ni2Co1MOF. At 10% rGO-Ni2Co1MOF, the highest specific capacitance value obtained was 14.36 F/g. This reveals that the presence of rGO in Ni2Co1MOF composite materials enhances the conductivity of conductive networks and the generation of active sites for reactions on the electrodes’ surfaces. Nevertheless, the minor increment in the specific capacitance value at 10% rGO-Ni2Co1MOF reveals that there is weak capacitive behavior in this material. A number of literature works conducted based on MOFs show that there are carriers that are localized in microporous MOFs, thus having limited capacitive behavior [56,57]. This reveals that the capacitive behavior is controlled by electron conduction and ion diffusion.

**Figure 16:**
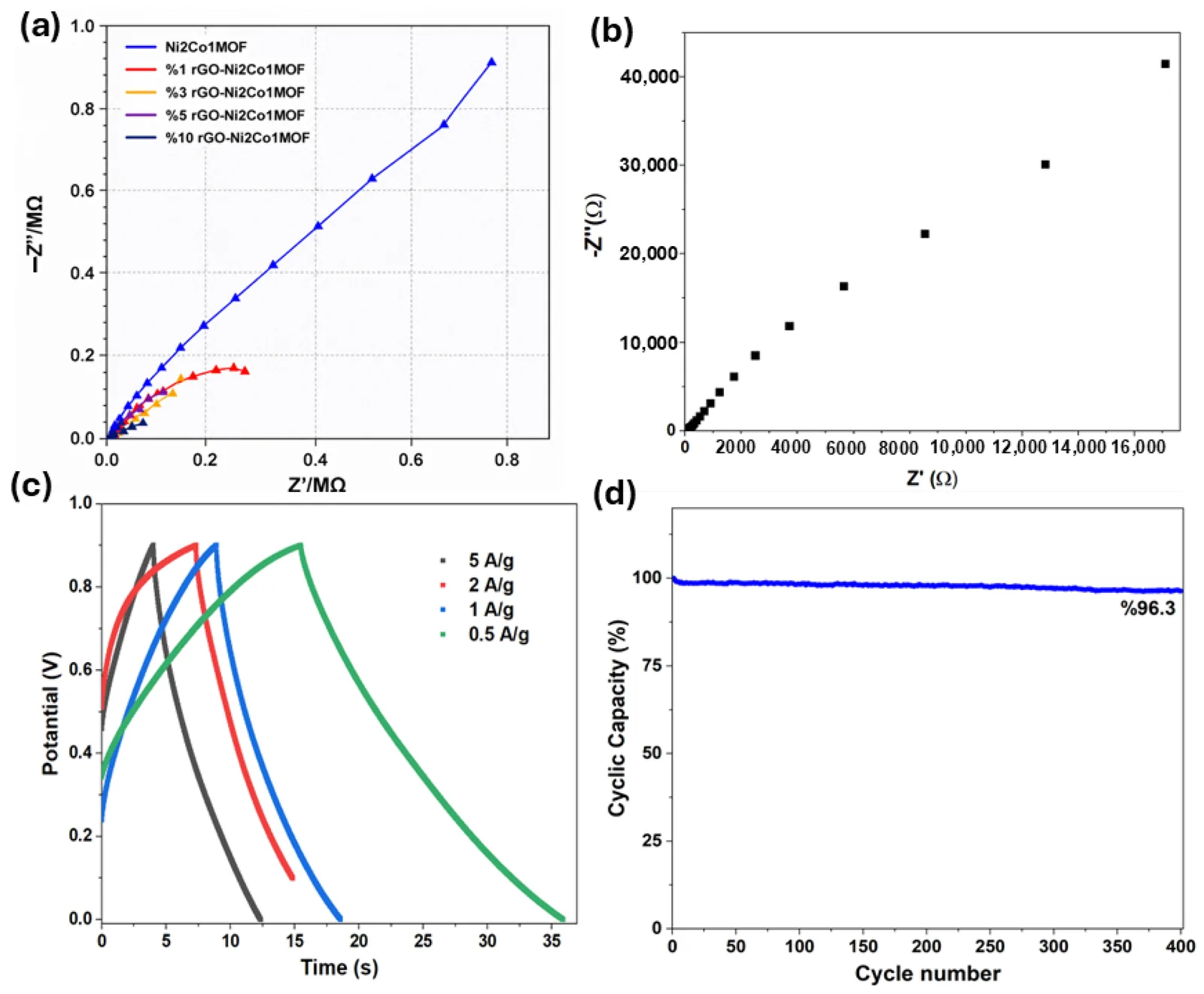
(**a**) Comparative impedance analysis results for Ni2Co1MOF and rGO-Ni2Co1MOF (1–10%) composites, (**b**) impedance analysis results for 10% rGO-Ni2Co1MOF, (**c**) GCD curves of 10% rGO-Ni2Co1MOF at different current densities, and (**d**) loop performance at 5 A g^−1^ current density (extended to 400).

Figure 16a,b displays both the impedance analysis data and charge/discharge curves for Ni2Co1MOF and rGO-Ni2Co1MOF (1–10%). Electrode kinetics data were obtained similarly to CV. These measurements have been performed in the range of 10 kHz to 100 kHz at the open-circuit potential. The sample of 10% rGO-Ni2Co1MOF, with the best electrochemical performance (current density, CV area, charge/discharge time), has very low internal resistance (R_s_ = 11.3 μ) and zero charge transfer resistance (R_ct_), since no separate circle can be seen in the Nyquist plot. Limited ion transport inside the pores implies that not only diffusion processes but also capacitive effects operate effectively [58]. The capacitive performance of the developed electrodes has been analyzed in detail via GCD measurements (Figure 16c,d). GCD curves have been registered at current densities in the range of 0.5 A g^−1^ to 5 A g^−1^ and at a potential value of 0.9 V. At a 0.5 A g^−1^ current density, deviations from EDLC behavior can be found on the GCD graphs, which perfectly corresponds to the CV results and shows the dominance of pseudocapacitive effects in the system [59]. This behavior is also consistent with the diffusion-dominant behavior observed in the EIS analysis.

##### Electrochemical Characterization of Ni1Co2MOF and rGO–Ni1Co2MOF (1–10 wt.%) Composites by CV, GCD, and EIS

**Figure 17:**
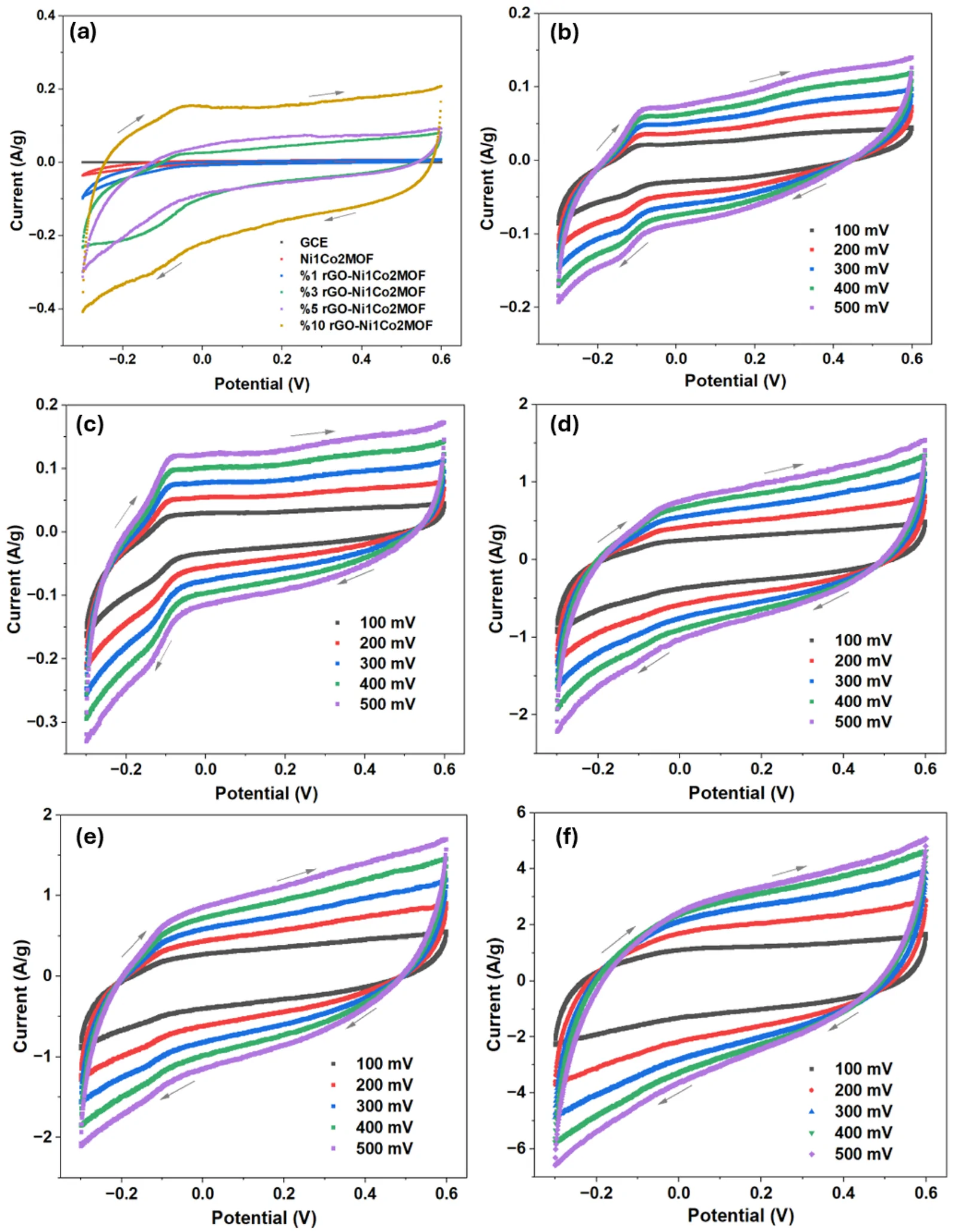
CV curves of GCE electrode, (**a**) Ni2Co1MOF, and rGO (1–10%)-Ni2Co1MOF composites at 10 mV s^−1^ scan rate and at different scan rates (100–500 mV s^−1^) for (**b**) Ni2Co1MOF, (**c**) 1% rGO-Ni2Co1MOF, (**d**) 3% rGO-Ni2Co1MOF, (**e**) 5% rGO-Ni2Co1MOF, and (**f**) 10% rGO-Ni2Co1MOF composites in 0.5 M HCl solution.

The cyclic voltammetry study of the Ni1Co2MOF and rGO-Ni1Co2MOF (1–10%) compounds was carried out in 0.5 M acid solution medium at varying scan rates of 10 to 500 mV s^−1^, and all graphs are presented in Figure 17. From the observation of the shape of the curve in the graph, it is clear that pseudocapacitance is more evident for Ni2Co1MOF compounds, with increased scan rates accompanied by increased current values and field broadening compared to the voltage range, implying that the electrochemical response was fast. Regarding the graphs presented above, it is clear that the CV graphs of the Ni2Co1MOF and rGO compounds were narrow when compared to those of the Ni1Co2MOF and rGO compounds at the same scan rates. The specific capacitance values of the Ni1Co2MOF compounds at a scan rate of 10 mV s^−1^ gradually increased with increasing rGO addition ratios, with 10% rGO-Ni1Co2MOF having a higher specific capacitance value (32.3 F/g).

**Figure 18:**
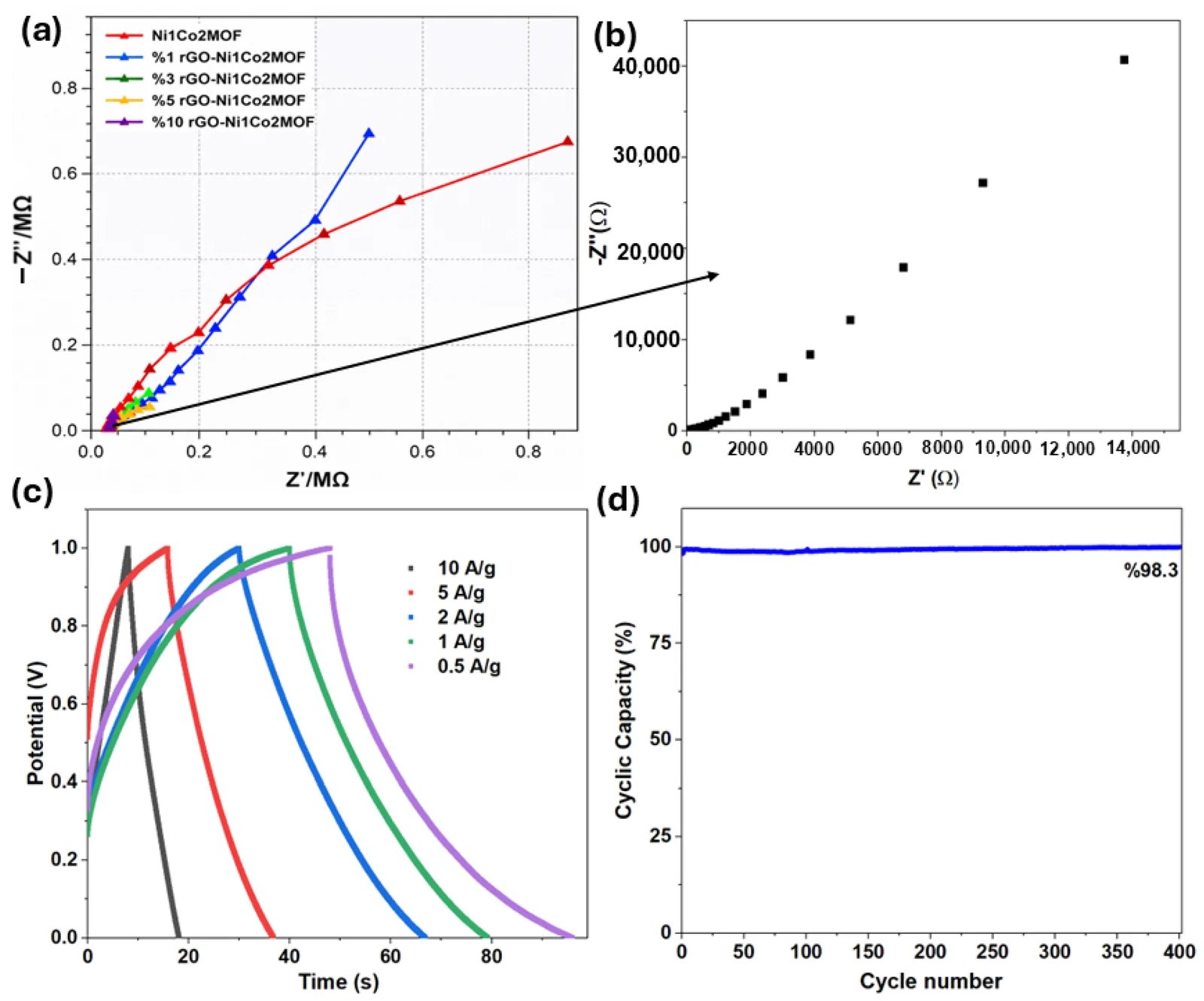
(**a**) Comparative impedance analysis results for Ni1Co2MOF and rGO-Ni1Co2MOF (1–10%) composites, (**b**) impedance analysis results for 10% rGO-Ni1Co2MOF, (**c**) GCD curves of 10% rGO-Ni1Co2MOF at different current densities, and (**d**) loop performance at 10 A g^−1^ current density (extended to 400).

In Figure 18, the impedance analysis results, along with the charge–discharge curves, for the Ni1Co2MOF and rGO-Ni1Co2MOF (1–10%) composites are illustrated to provide insight into the electrode kinetics. The experiments were performed at the open-circuit potential within the frequency range of 10 kHz-100 kHz. It can be seen that the rGO-Ni1Co2MOF 10% composite demonstrates the best electrochemical performance, with a high current density, CV area, and charge/discharge time. The presence of internal resistance in this composite is negligible (R_s_ = 11.1 μ), and there is no distinct circle in the Nyquist plot; thus, the charge transfer resistance is negligible (R_ct_). Thus, it can be said that rGO-Ni1Co2MOF composites have similar features to Ni2Co1MOF and rGO-Ni2Co1MOF composites. The low ion transport in the porous structure of this system proves that diffusion-controlled processes and capacitive mechanisms work effectively [58]. To study the capacitive characteristics of the prepared electrodes, the GCD technique was used (Figure 18c,d). GCD curves were recorded for different values of current density (0.5 A g^−1^–10 A g^−1^) at a 0.9 V potential. Deviations from the EDLC characteristic were found in the GCD curves; they were in accordance with the results of the CV analysis. Thus, pseudocapacitive processes affect the system [59]. The increase in the quantity of cobalt in the composite led to an increase in Csp and the charge/discharge time. An increase in the rGO amount decreased the internal resistance [60] and enhanced the conductivity in the HCl electrolyte. After 400 cycles, 98% and 96% of the values were preserved for the 10% rGO-Ni1Co2MOF and 10% rGO-Ni2Co1MOF, respectively.

### 4.7. Effects of In Situ Photopolymerization on Electrochemical Conductivity of MOF-Based Composites

#### 4.7.1. Electrochemical Capacitance Behavior of Ni-Co MOFs (Ni/Co = 2:1–1:2) and rGO–MOF Composites (1–10 wt.%)

**Figure 19:**
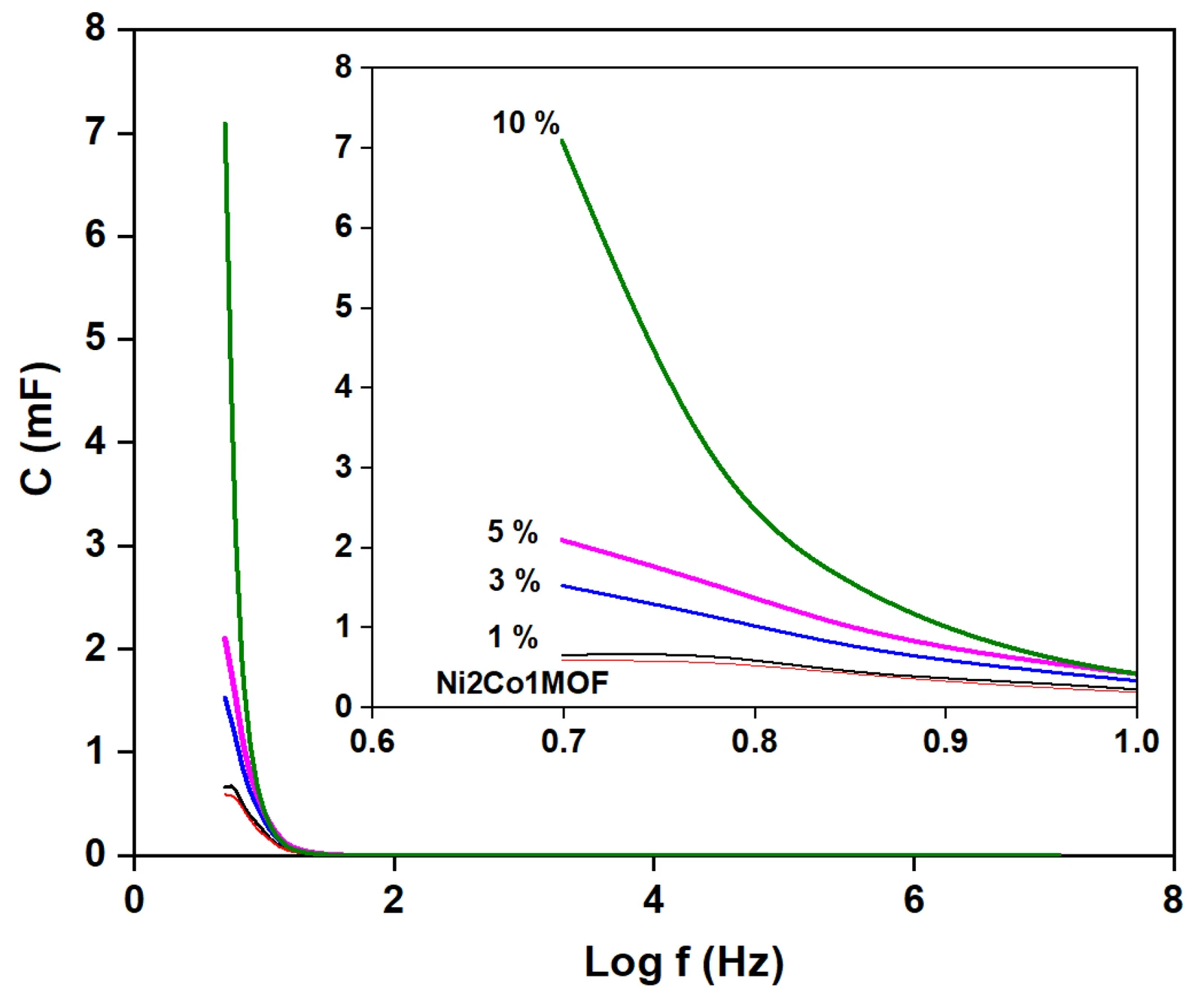
Frequency-dependent capacitance changes of Ni2Co1MOF and rGO (1–10%)-Ni2Co1MOF composites.

The frequency-dependent capacitance variation graphs of the Ni2Co1MOF and rGO (1–10%)-Ni2Co1MOF composites are given in Figure 19. It is observed that both the Ni2Co1MOF and 1–10% rGO-Ni2Co1MOF composites exhibit high capacitance that increases with decreasing frequencies but decreases significantly with increasing frequencies. The reason for the decrease in capacitance is hypothesized to be the capacitive effect caused by the accumulation of charges at the interface, particularly in materials with different electrical characteristics, known as Maxwell–Wagner polarization. This effect manifests itself at low frequencies but fails to adapt to increasing frequencies. It is clearly seen that the capacitive property of Ni2Co1MOF materials is increased approximately tenfold with increasing rGO content, meaning that increasing the rGO percentage reduces the polarization loss and increases the effectiveness of interfacial polarization.

**Figure 20:**
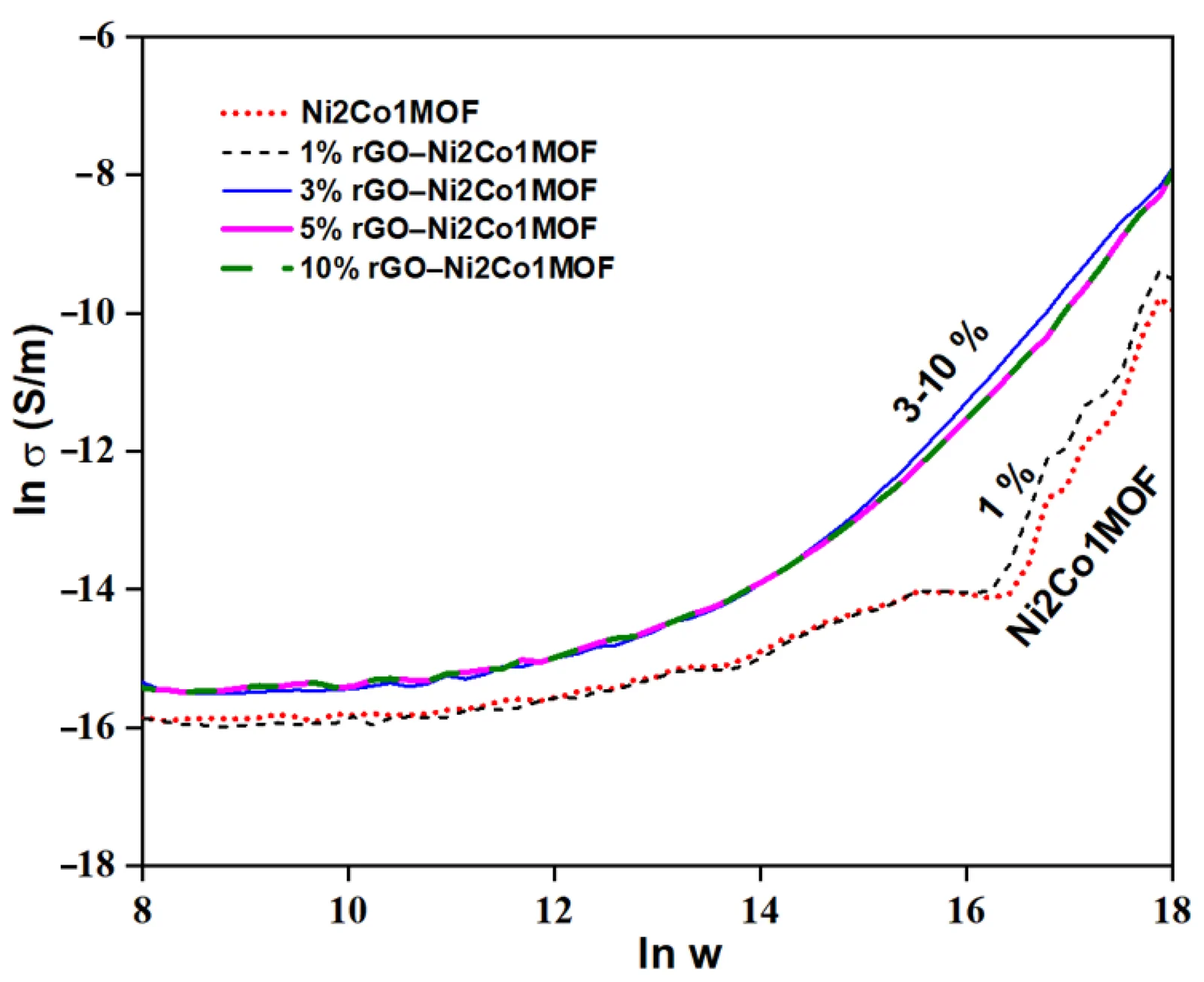
Frequency-dependent conductivity variation of Ni1Co2MOF and rGO (1–10%)–Ni2Co1MOF composites.

The frequency-dependent conductivity graph shown in Figure 20 clearly demonstrates that the frequency dependence of the conductivity consists of DC-like and AC conductivity regions; constant conductivity appears for all materials in the low-frequency range [61]; and, as the frequencies increase, there is an increase in conductivity following the power law behavior described by [62], with conduction occurring via a barrier-controlled jumping mechanism [63]. After the addition of rGO 3% and above, the conductivity of these materials increases (~2 times) [64] to an approximately constant value.

**Figure 21:**
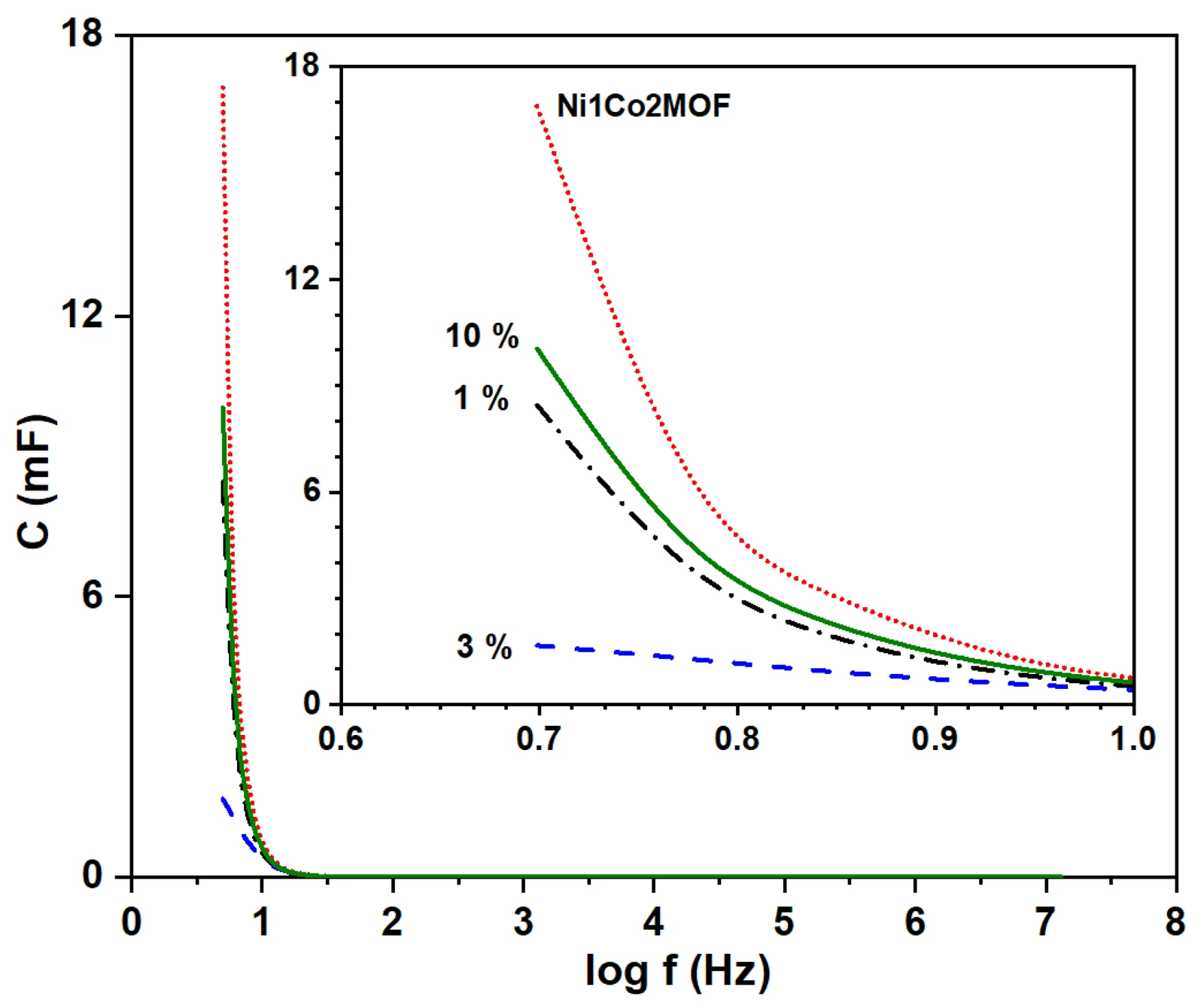
Frequency-dependent capacitance changes of Ni1Co2MOF and rGO (1–10%)–Ni1Co2MOF composites due to dielectrics/polarization.

In Figure 21, a high capacitance level is present for the Ni1Co2MOF (1:2) and rGO-Ni2Co1MOF composites in the same low-frequency range as for the 2:1 ratio; however, the decrease is sharper with increasing frequencies compared to the 2:1 Ni2Co1MOF material due to the inability to achieve space charge accumulation, dipole orientation, and interface polarization in order to respond quickly to changes in the electric field as the frequency increases. Due to localized charges formed within the structure and contributing to the polarization process, the material with the highest capacitance level is Ni1Co2MOF, including Ni1Co2MOF and those with rGO. Although the increase is not linear with increasing rGO, the combination of Ni1Co2MOF with rGO is comparable to Ni2Co1MOF without rGO in terms of capacitance, and its decrease at high frequency levels is much smoother than in the other two samples. It was also observed that the lowest capacitance level was exhibited by Ni1Co2MOF containing 3% rGO, which was associated with the higher mobility of charge carriers and less localized charge accumulation. Furthermore, the non-homogeneity in the distribution of rGO indicates that it leads to the aggregation of the material and thus negatively affects interface polarization.

**Figure 22:**
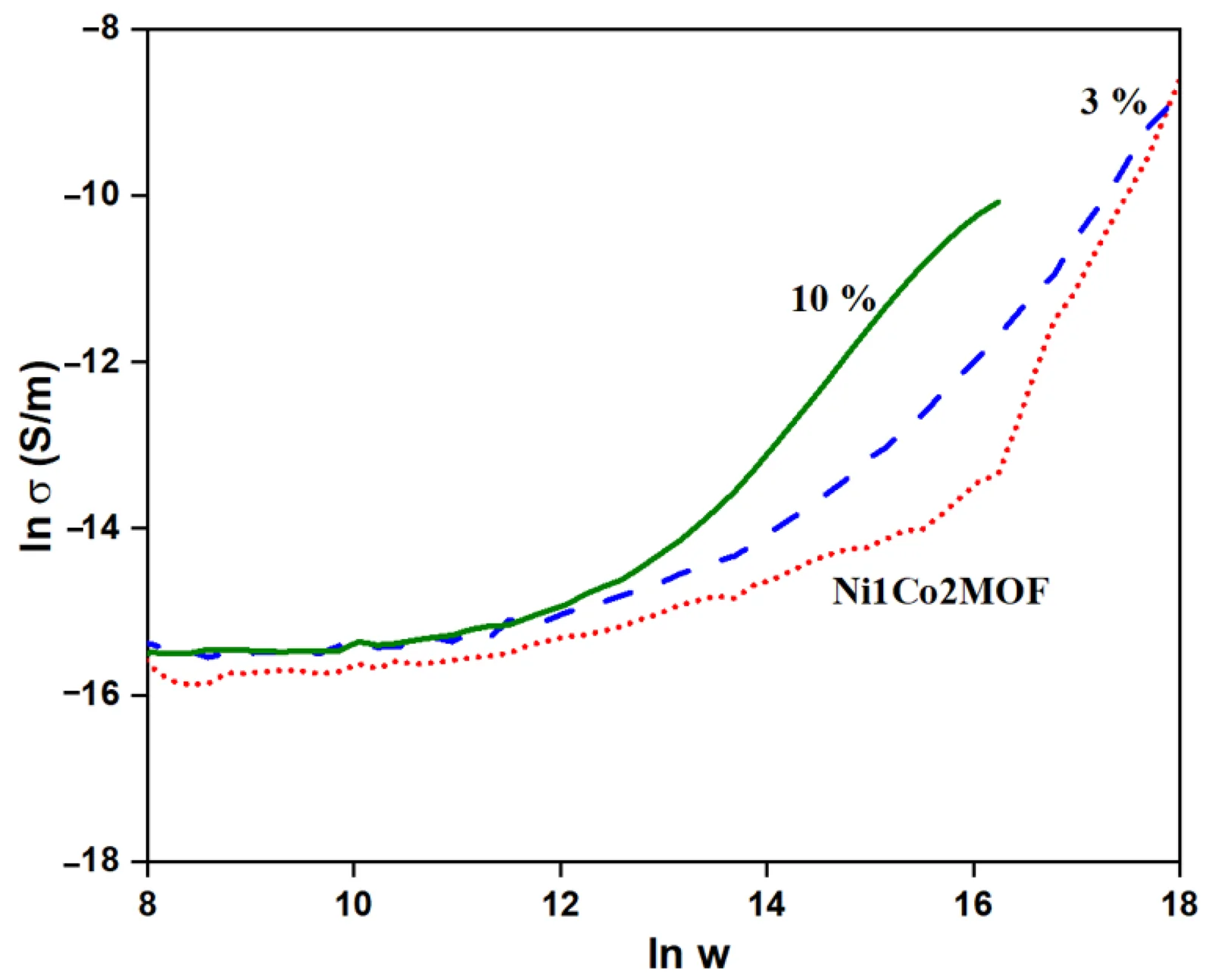
Frequency-dependent conductivity changes of Ni1Co2MOF and rGO (3 and 10%)–Ni1Co2MOF composites.

Figure 22 shows the frequency dependence graph of the AC conductivity for the Ni1Co2MOF and rGO–Ni1Co2MOF composites. The materials exhibit a two-zone characteristic similarly to that seen in Figure 20 for Ni2Co1MOF [65], and the AC conductivity onset frequency is approximately the same for Ni2Co1MOF, while it shifts to lower frequencies with rGO inclusion, possibly due to inhomogeneity compared to the 2:1 ratio. It can be said that it shows similar characteristics to those defined by the classical Jonscher power law, with an n > 1 relationship. Increasing the amount of rGO inclusion resulted in a multifold increase in conductivity, which was not as systematic as in the 2:1 material. The highest conductivity was found for the Ni1Co2MOF sample with 3% rGO, while those containing higher amounts exhibited significantly lower conductivity due to clustering at the 1:2 ratio. Therefore, the absence of free electrons in the MOF/rGO system indicates that the main feature within the system is localization and that rGO addition becomes optimum when a certain amount of rGO addition is reached, contributing to the formation of conductive networks [66]. The slope being less than one in the high frequency range may indicate that conductivity occurs due to a jump process with barrier control [63]. The maximum values of capacitance and conductivity were observed in the 1:2 and 2:1 NiCoMOF materials at different rGO inclusion amounts of 3% and 10%, respectively. It is understood that the active Ni and Co metals added to the MOF during production act as key centers in material formation, creating pseudocapacitive formation [67], and this process can be controlled with rGO addition along with the Ni/Co ratio.

These observations are also consistent with recent studies demonstrating that the optimization of the electronic structure and interfacial charge transfer pathways is essential for improving electrochemical performance by facilitating charge transfer and accelerating redox kinetics in advanced functional materials [68].

#### 4.7.2. Dielectric Properties of UV-Cured PEGMEA/PEGDA/MOF and PEGMEA/PEGDA/rGO–MOF Thin Films

**Figure 23:**
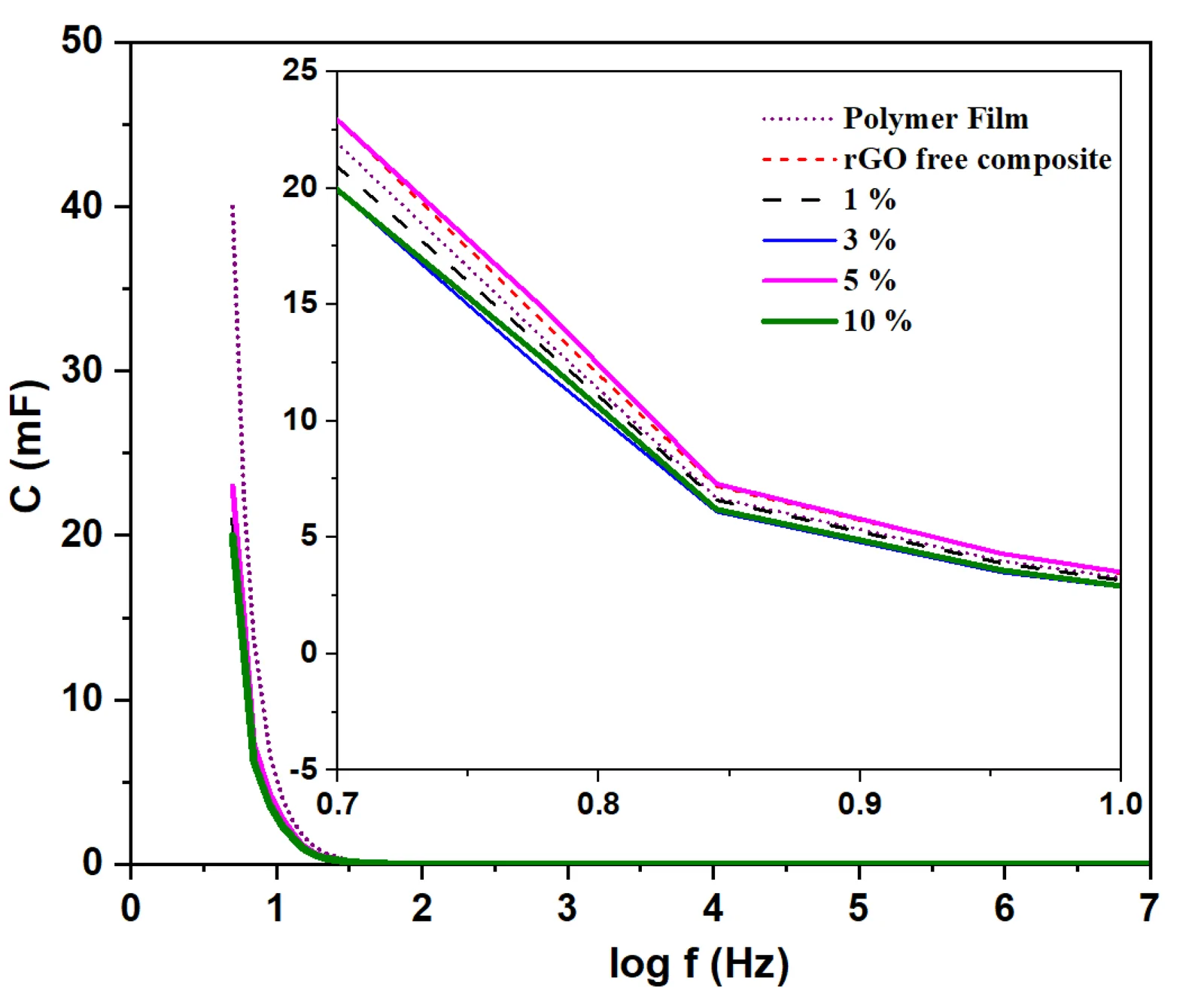
Frequency-dependent capacitance variations of polymer film, rGO free, and rGO (1–10%) polymeric composites of Ni2Co1MOF.

The frequency-dependent capacitance changes of the PEGMEA/PEGDA/MOF and PEGMEA/PEGDA/MOF/rGO composite thin films are given in Figure 23.

As illustrated in Figure 23, it can be seen that all polymer composite films show identical trends, with the capacitance reducing systematically with an increase in frequency. The marginal variation in the graphs suggests that the incorporation of rGO does not lead to considerable variation in the behavior of the system within the given frequency range. The low value of capacitance recorded may be attributed to filler agglomeration and interface heterogeneity.

**Figure 24:**
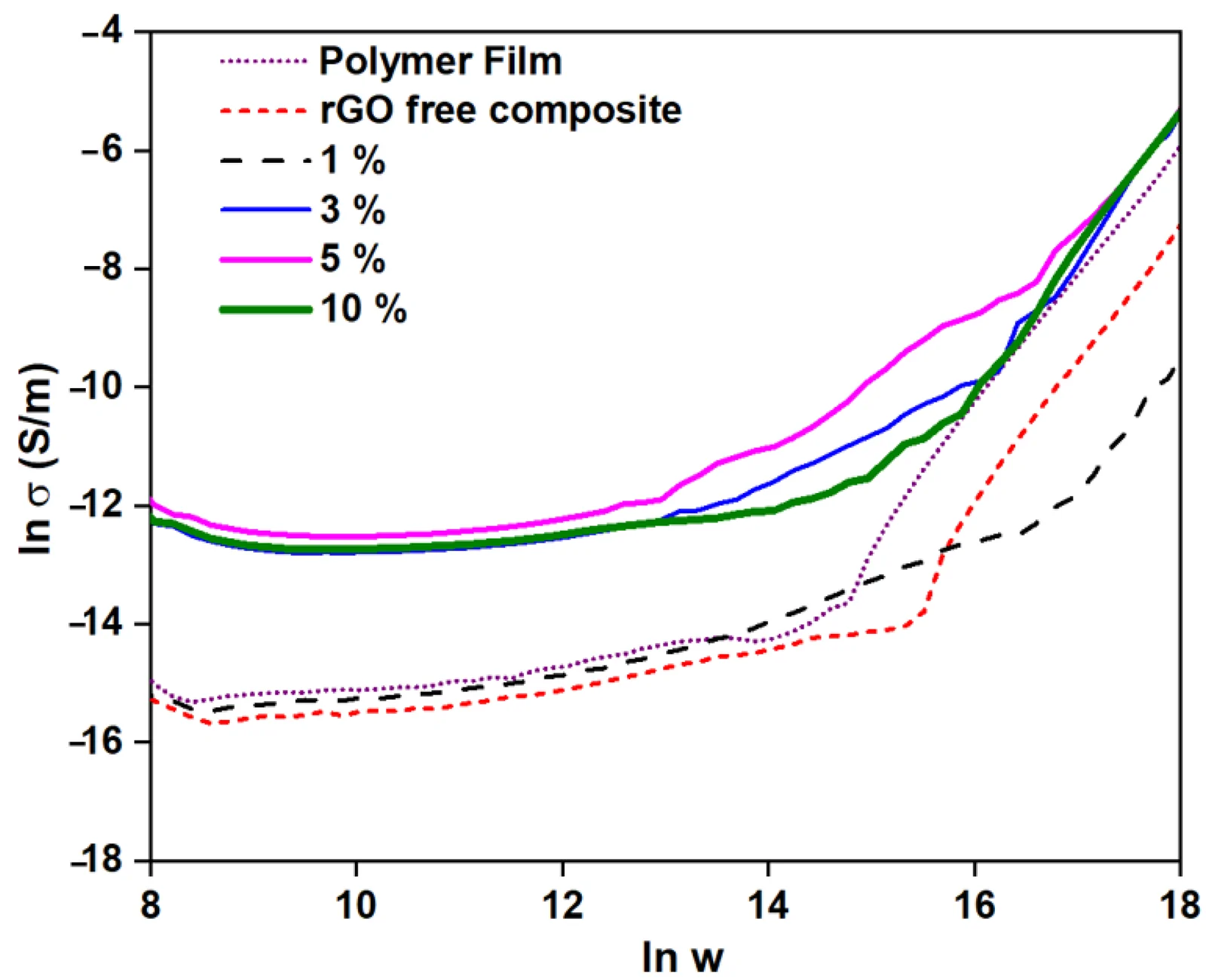
Frequency-dependent conductivity variations of polymeric film, rGO free, and rGO (1–10%) polymeric composites of Ni2Co1MOF.

From the frequency-dependent graphs of the conductivity (Figure 24), it can be seen that, in the low-frequency region, all samples have constant DC conductivity independently of the frequency, whereas the conductivity shows a marked increase in the high-frequency region. This implies that the samples under consideration do not behave according to the conventional hopping mechanism based on Jonscher’s power law because, here, n > 1.

It has been revealed that the creation of a conductive network within the composite structure is favored by rGO addition, and the conductivity is greatly enhanced, particularly in the high-frequency range. The composite structure with greater than 3% rGO addition has better conductivity (about 2 × 10^2^ times) at lower frequencies, giving rise to the minimum resistance and activation energy. However, on the other hand, the capacitance-decreasing effect of rGO addition suggests that the increment in the number of interfaces can affect carrier transport at lower frequencies. It thus can be said that the optimal addition ratio is crucial when considering an increment in conductivity with rGO addition, and in the event of excessive addition, conduction could be hindered due to interface-related barrier effects. Beyond this, the conductivity characteristics obtained in this study indicate that composites of this nature may be well suited for integration into sensor-based applications [69,70,71]. Although Maxwell–Wagner polarization operates under low-frequency conditions, the sharp drop at high frequencies suggests that the effect of the polarization becomes less effective. The PEGMEA/PEGDA polymer film possesses high dipole strength, which gives it the highest capacitance at low frequencies.

**Figure 25:**
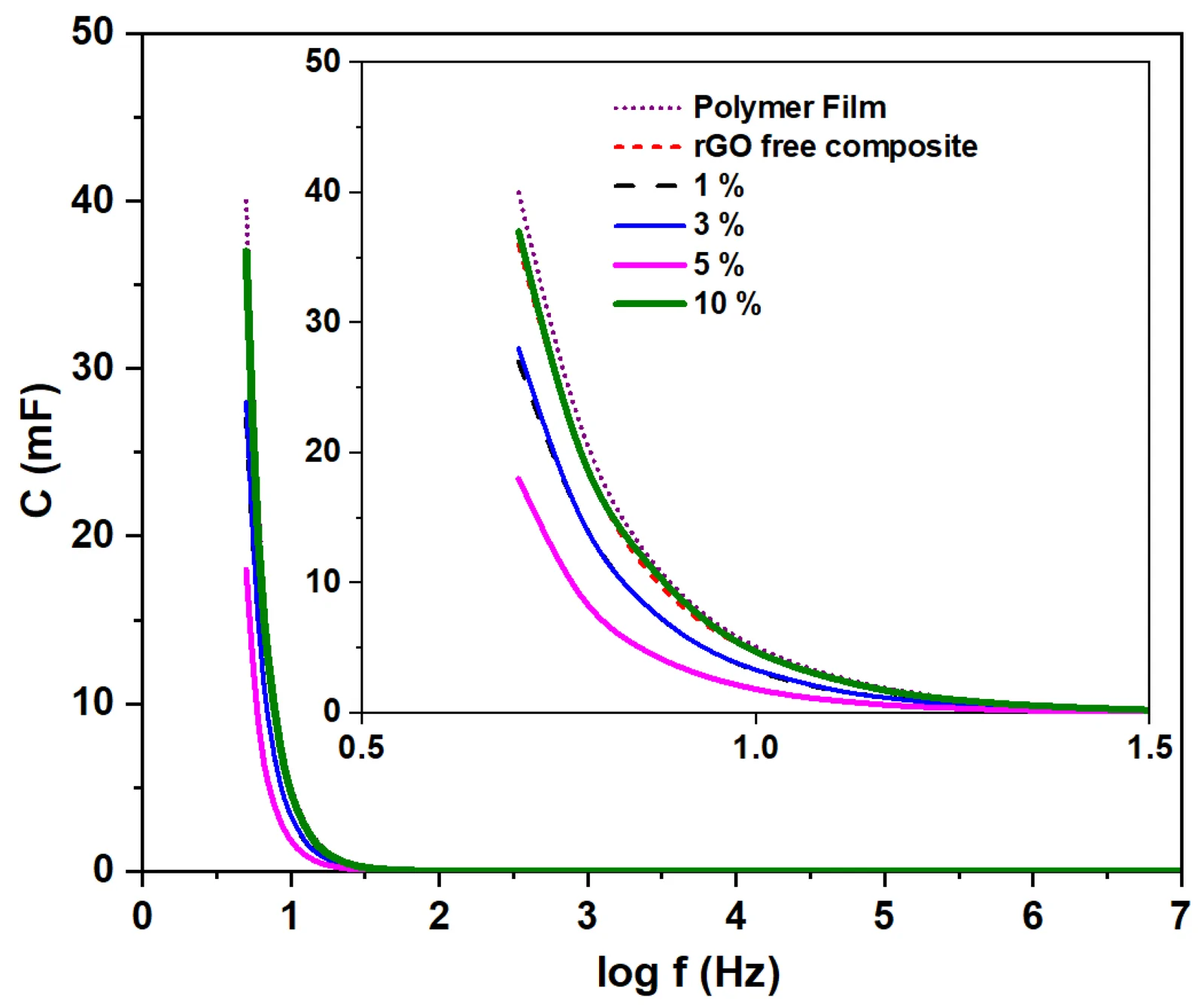
Frequency-dependent capacitance changes of polymeric composites of Ni1Co2MOF and rGO (1–10%)–Ni1Co2MOF composites.

When Ni1Co2MOF is introduced into the polymer film, there is a slight drop in capacitance; however, no linear relationship is seen as more rGO is added (Figure 25). Due to the introduction of rGO, charge accumulation and dipole addition become lower, while Maxwell–Wagner polarization becomes higher.

**Figure 26:**
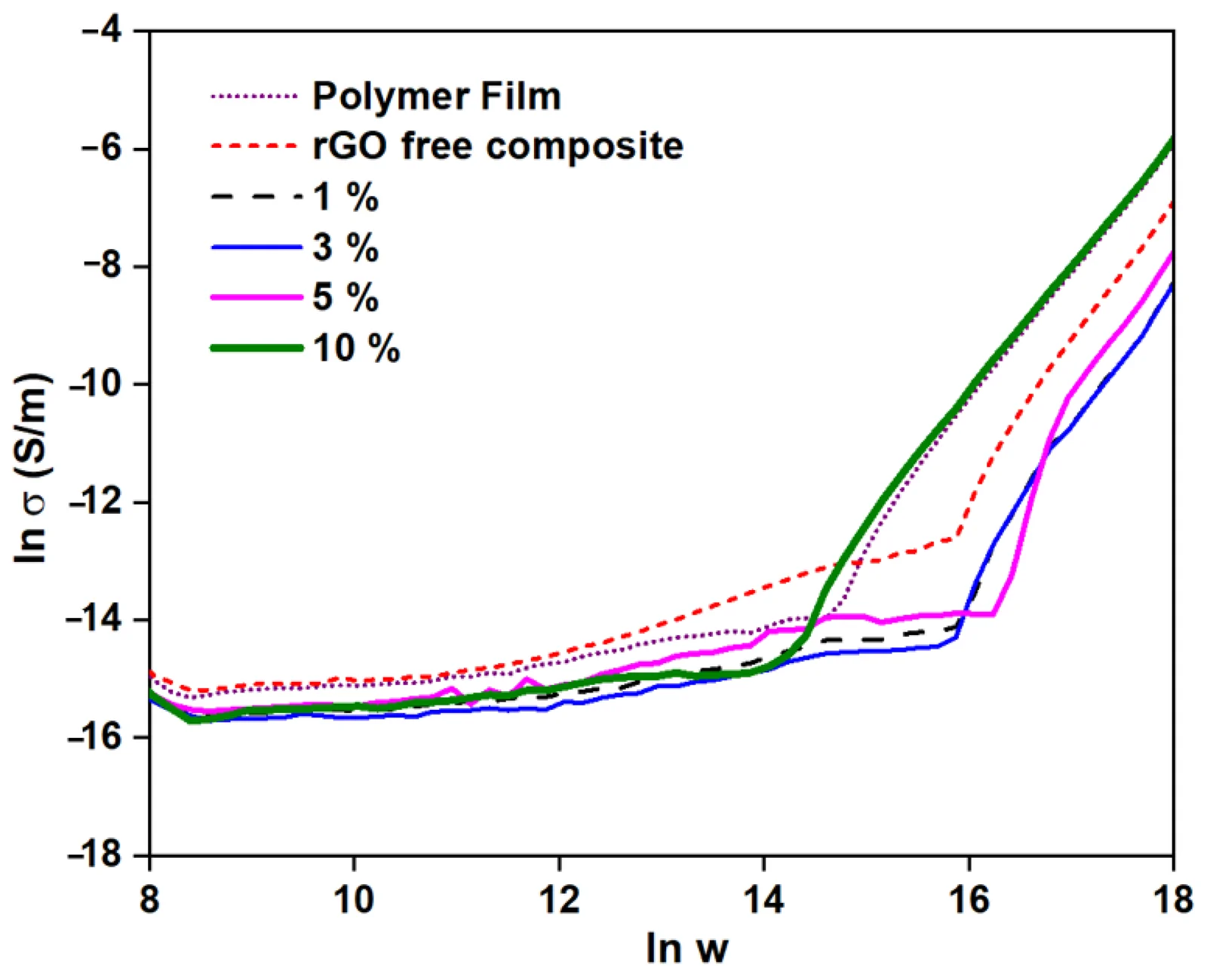
Frequency-dependent conductivity variations of polymeric composites of Ni1Co2MOF and rGO (1–10%)–Ni1Co2MOF composites.

From Figure 26 above, a detailed analysis shows that the material possesses frequency-independent DC conductivity at low frequencies. However, at high frequencies, the material exhibits increased conductivity, implying that it follows the Jonscher power law, with its carriers moving via hopping conduction with n > 1 values.

This research showed that the presence of rGO leads to an improvement in conductive network formation and consequently increases the conductance, especially in the high-frequency region. It can be clearly observed that the sample with a concentration of 3% shows the lowest resistivity and activation energy and thus higher conductance at low frequencies. However, even though the sample with a concentration of 10% demonstrates an increase in conductivity at high frequencies, it is expected that carrier mobility may be limited at low frequencies due to an increase in interfaces. From these observations, it can be concluded that optimal addition is crucial for improving the conductivity of samples and that, if the rGO level exceeds certain values, interface barriers might affect the conduction process.

Furthermore, the conductivity behavior observed in this study suggests that such composite materials may hold significant potential for application in sensing technologies [69,70,71]. Jonsher’s equation, where n > 1, implies that there is more than one process involved, unlike the traditional hopping process, since interface changes occur at the composite triple point of the polymer, rGO, and MOF. Maxwell–Wegner–Sillars interface polarization can be appropriately applied to describe both the capacitive behavior and conducting process for low frequencies. From an analysis of the conductivity graph, we note that the AC conductivity increases with increasing frequencies and an interactive process occurs at the composite triple interface.

The introduction of MOF fillers into polymers, especially UV-curable acrylate-based systems, faces challenges associated with filler agglomeration and poor compatibility at the polymer–filler interface. Both issues significantly impact the thermal, dielectric, and mechanical characteristics of the obtained composite materials. Despite the growing interest in introducing rGO/MOF hybrid fillers into UV-curable acrylate systems, very few studies have comprehensively examined their effects on the dielectric characteristics and Maxwell–Wagner–Sillars (MWS) interfacial polarization. As demonstrated in the case of EP/ZIF-8/rGO hybrid composites [72], the addition of conductive nanofillers can efficiently regulate charge transport within the polymer matrix without deteriorating its mechanical and dielectric properties. The large interfacial area formed by the rGO/MOF hybrid increases the number of polarization centers and substantially enhances the dielectric constant compared with a pure acrylate matrix. On the other hand, poor dispersion, interfacial voids, and filler agglomeration facilitate uncontrolled space-charge migration, leading to increased leakage currents and higher dielectric losses (tan δ). Therefore, achieving the homogeneous dispersion of the rGO/MOF hybrid within the polymer matrix is essential for maximizing interfacial polarization while maintaining low dielectric losses.

In the current research, the UV-cured PEGMEA/PEGDA composites based on rGO/MOF hybrids showed a distinctive percolation threshold at rGO content equal to 3 wt%. Such a composition demonstrated the most balanced thermal, dielectric, and electrical characteristics. Moreover, 3 wt% rGO slightly decreased the value of Tg, demonstrating an increase in the mobility of the polymer chains and flexibility while suppressing filler agglomeration and facilitating the formation of a conductive network. As a result, this composite showed the highest stability in terms of the dielectric and electrical characteristics among all investigated compositions.

On the other hand, an increase in the amount of rGO to 10 wt% and higher caused excessive filler loading, which led to the restacking and agglomeration of the graphene nanosheets. Such phenomena increased the interfacial resistance, disrupted the continuity of the conductive network, and decreased the positive impact of interfacial polarization. As a result, excessive rGO loading had negative effects on the dielectric characteristics of the composites, despite the higher concentration of the conductive filler.

#### 4.7.3. Thermal and Morphological Characterization of UV-Cured PEGMEA/PEGDA/MOF and PEGMEA/PEGDA/rGO–MOF Thin Films

Monomer mixtures based on poly(ethylene glycol) methyl ether acrylate (PEGMEA) and poly(ethylene glycol) diacrylate (PEGDA) are attractive materials for flexible sensors, conductive membranes, and smart coatings owing to their excellent flexibility, moisture uptake, and biocompatibility. However, their intrinsic electrical conductivity and mechanical stability remain insufficient for many advanced applications. To overcome these limitations, metal–organic frameworks (MOFs) have emerged as promising functional fillers for polymer matrices because of their tunable porous structures, large specific surface areas, and the ability to tailor the chemical compositions of their metal centers. The incorporation of MOFs into polymer matrices can significantly enhance the electrical, thermal, and morphological properties of the resulting composite materials.

Nevertheless, bimetallic Ni-Co MOFs provide a wider electrochemical window and higher active center density than monometallic MOFs by taking advantage of the redox behavior of Ni^2+^/Ni^3+^ and Co^2+^/Co^3+^ ion pairs. The key limitation in introducing pure MOFs into the polymer matrix is associated with the tendency for self-agglomeration and poor interfacial compatibility between the metal oxides. In contrast, rGO ensures the uniform dispersion of the MOF particles and the creation of a barrier network that prevents thermal degradation due to its high electron transportation capacity and surface functionality at the nanometer level.

The thermal and morphological properties of UV-cured PEGMEA/PEGDA composite films containing Ni2Co1MOF, Ni1Co2MOF, and their corresponding rGO–MOF composites (3 and 10 wt.% rGO) were investigated by thermogravimetric analysis (TGA), differential scanning calorimetry (DSC), and scanning electron microscopy (SEM). The results were analyzed and discussed in relation to the compositions of the incorporated fillers.

A total of eight sample groups were examined. UV-cured PEGMEA/PEGDA served as the reference material, while the remaining seven formulations were prepared by incorporating NiCoMOFs and rGO–NiCoMOF composites with two different Ni/Co molar ratios (2:1 and 1:2) and two rGO loadings (3 and 10 wt.%). The investigated samples were as follows:

- UV-cured PEGMEA/PEGDA (reference);
- UV-cured PEGMEA/PEGDA/Ni2Co1MOF;
- UV-cured PEGMEA/PEGDA/3 wt.% rGO–Ni2Co1MOF;
- UV-cured PEGMEA/PEGDA/10 wt.% rGO–Ni2Co1MOF;
- UV-cured PEGMEA/PEGDA/Ni1Co2MOF;
- UV-cured PEGMEA/PEGDA/3 wt.% rGO–Ni1Co2MOF;
- UV-cured PEGMEA/PEGDA/10 wt.% rGO–Ni1Co2MOF.

**Figure 27:**
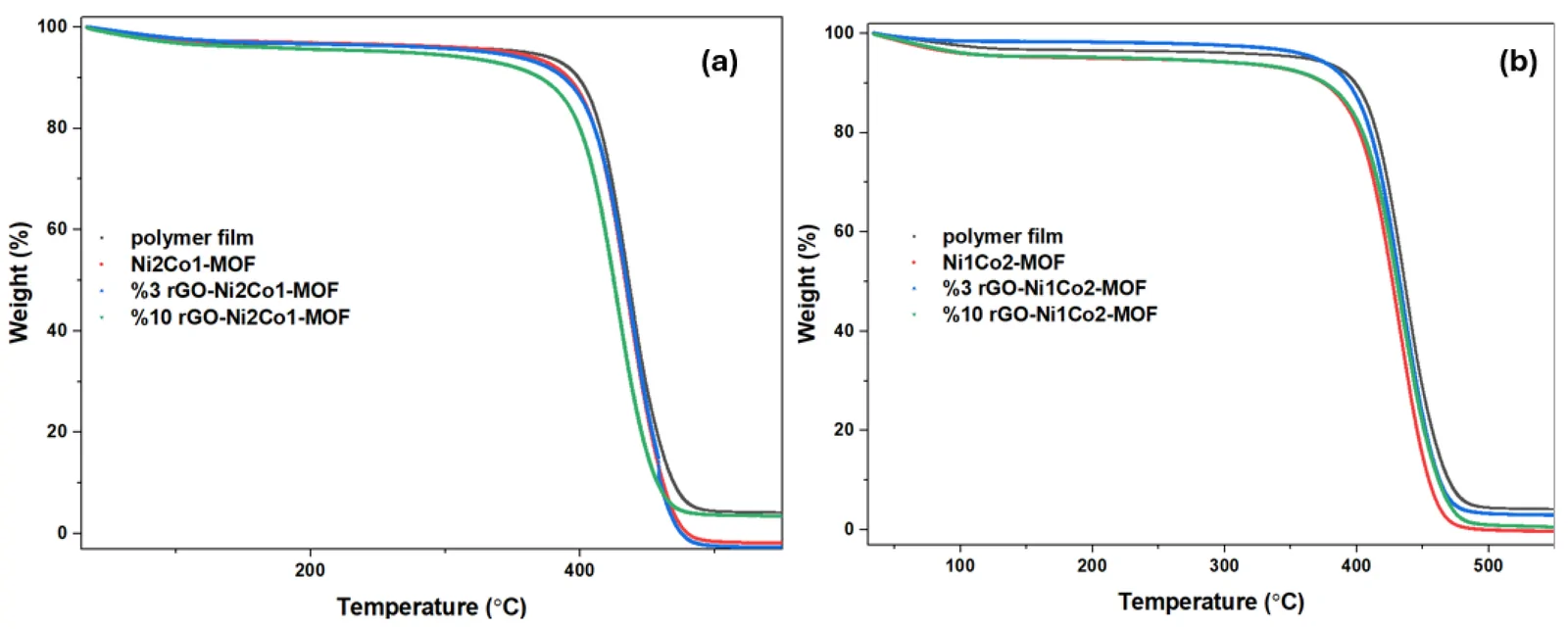
TGA curves of the neat PEGMEA/PEGDA film and PEGMEA/PEGDA composite films containing (**a**) Ni2Co1MOF, 3 wt.% rGO–Ni2Co1MOF, and 10 wt.% rGO–Ni2Co1MOF and (**b**) Ni1Co2MOF, 3 wt.% rGO–Ni1Co2MOF, and 10 wt.% rGO–Ni1Co2MOF, illustrating the influence of the MOF composition and rGO loading on the thermal degradation behavior and thermal stability of the polymer composites.

Figure 27 presents the TGA thermograms of the UV-cured PEGMEA/PEGDA film and the corresponding Ni2Co1MOF, 3 wt.% rGO–Ni2Co1MOF, and 10 wt.% rGO–Ni2Co1MOF-containing composite films. Similar analyses were performed for the Ni1Co2MOF series.

Figure 27a compares the thermal degradation behavior of the neat PEGMEA/PEGDA film and the Ni2Co1MOF-containing composites. All samples exhibit a similar single-step degradation process, indicating that the incorporation of the MOF does not fundamentally alter the degradation mechanism of the polymer matrix. The neat polymer begins to degrade at approximately 370 °C, whereas the incorporation of Ni2Co1MOF slightly decreases the onset degradation temperature to about 355 °C. The incorporation of 3 wt.% rGO results in a slight improvement in thermal stability, shifting the degradation onset to approximately 380 °C, whereas increasing the rGO content to 10 wt.% provides no further improvement. This behavior is attributed to the barrier effect of well-dispersed graphene sheets at low loading and their partial aggregation at higher loading. The residual mass at high temperatures remains comparable for all samples, indicating similar inorganic content after thermal decomposition.

Figure 27b presents the TGA curves of the Ni1Co2MOF-based composites. Similarly to the Ni2Co1MOF series, all samples display nearly identical degradation profiles, suggesting that the Ni/Co ratio has only a minor influence on the thermal decomposition behavior of the polymer composites. The addition of 3 wt.% rGO slightly delays the onset of degradation, whereas increasing the rGO content to 10 wt.% does not provide a significant additional improvement. The small differences in the residual mass are attributed to the formation of thermally stable metal oxide residues after decomposition.

Overall, the TGA results demonstrate that the incorporation of Ni-Co MOFs has only a limited influence on the thermal stability of the UV-cured PEGMEA/PEGDA matrix. The degradation behavior of all composites remains very similar to that of the pristine polymer, while the addition of a small amount of rGO (3 wt.%) provides a modest improvement in thermal stability. Increasing the rGO content beyond this level does not further enhance the thermal resistance, indicating that excessive rGO loading does not produce an additional barrier effect.

**Figure 28:**
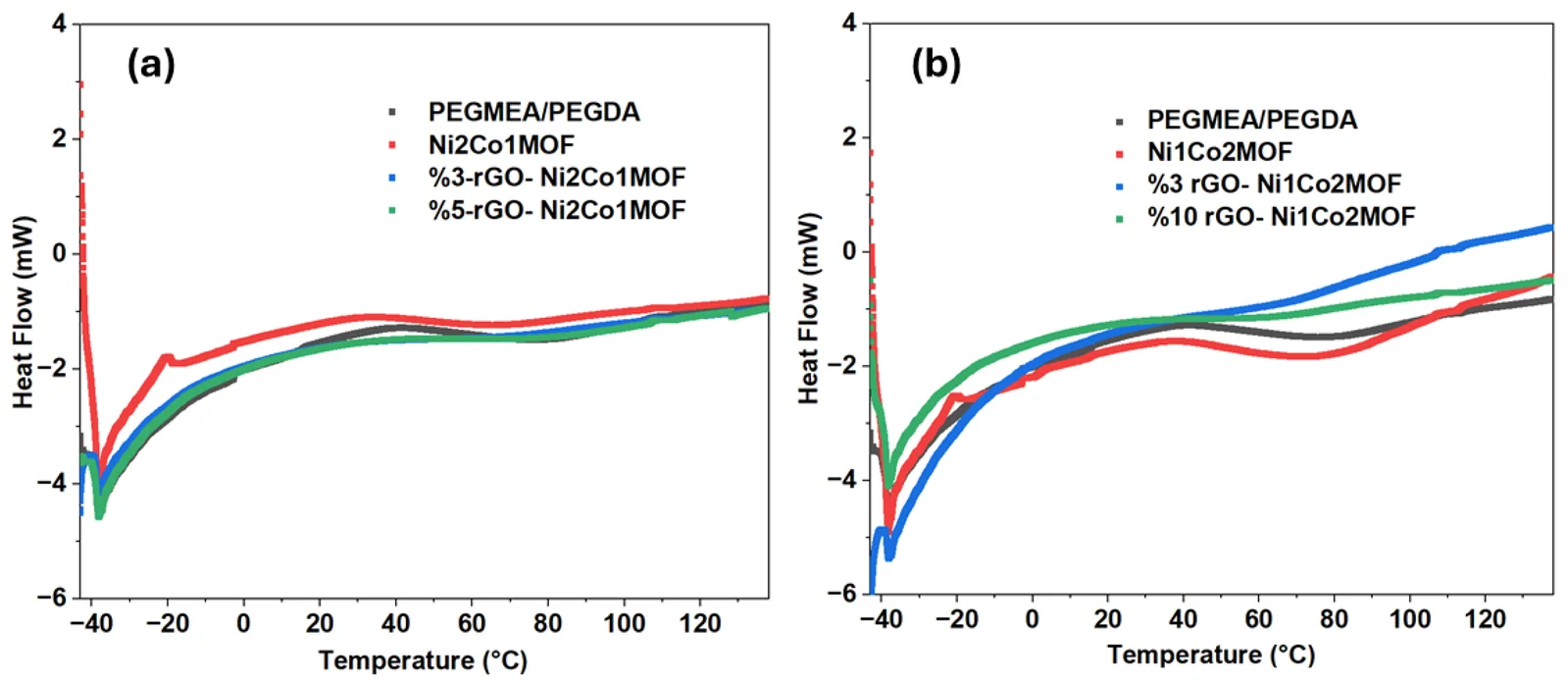
DSC thermograms of the neat PEGMEA/PEGDA film and PEGMEA/PEGDA composite films containing (**a**) Ni2Co1MOF, 3 wt.% rGO–Ni2Co1MOF, and 10 wt.% rGO–Ni2Co1MOF and (**b**) Ni1Co2MOF, 3 wt.% rGO–Ni1Co2MOF, and 10 wt.% rGO–Ni1Co2MOF, illustrating the influence of the MOF composition and rGO loading on the glass transition behavior of the polymer composites.

Figure 28a depicts DSC heat flow temperature curves for the Ni2Co1MOF series and the Ni1Co2MOF series. DSC measurements were carried out from about −45 °C to +140 °C. A glass transition area (Tg) can be identified in the DSC curves of both series within the range of about −35 °C to −25 °C. This means that temperatures within the range of −60 °C to −20 °C, which are typical for PEG-based acrylic polymers, occur in the DSC curve. According to the data from the previous experiments, it can be concluded that PEG-based polymers have large free volumes and chain mobility. The influences of MOFs and rGO additives on polymer chain mobility can be seen in the DSC curves.

It was found that the glass transition temperature (Tg) of the PEGMEA/PEGDA system was −38 °C. Moreover, the Tg was determined for a composite including Ni2Co1MOF, which was equal to −38 °C. When the Ni2Co1MOF/PEGMEA/PEGDA composite included 3% of rGO, the Tg increased slightly to −37 °C, while it was obtained as −39 °C in the case of 10% rGO addition. The Tg temperature of a composite containing Ni1Co2MOF was determined as −38 °C. At 3% rGO in the Ni1Co2MOF/PEGMEA/PEGDA composite, the Tg temperature was measured as −37.5 °C, while, at 10% rGO content, the Tg amounted to −38.2 °C.

Thus, it can be concluded that the addition of rGO causes only minor changes in the Tg of the composite. In particular, the addition of 3% rGO to a system based on Ni2Co1MOF shifted the Tg slightly from −38 °C to −37 °C, whereas increasing the rGO content to 10% resulted in a Tg of −39 °C. Similar small variations were observed for the Ni1Co2MOF-based composite (Figure 28b), where the Tg changed from −38 °C to −37.5 °C at 3% rGO and to −38.2 °C at 10% rGO. Overall, the observed Tg variations were within approximately 1 °C, indicating that rGO incorporation has only a limited influence on the segmental mobility of the PEGMEA/PEGDA matrix. Moreover, the similarity in the Tg values of the composites with different MOFs demonstrates that the Ni/Co ratio does not significantly affect the Tg, unlike the quantity of rGO [73,74].

Summing up the above results, the DSC results indicate that the incorporation of rGO does not substantially alter the glass transition behavior of the composites. Although only slight changes in Tg were observed, the presence of rGO may still contribute to improved interfacial interactions and enhanced thermal stability, as confirmed by the TGA results. Within the above framework, rGO-filled hybrids deserve attention due to their ability to create a larger interface zone and modify the dynamics of polymer chains compared with particulate fillers. Combining rGO and MOF zones may contribute to building a better-structured internal arrangement, which enhances interfacial interactions. Such an interpretation is consistent with the enhanced thermal behavior of the composites detected by TGA and supports the formation of a more ordered polymer microenvironment. From the viewpoint of the existing studies in the GO/MOF literature, such interactions may result in the greater stability and electrical conductivity of the composite [64,75].

**Figure 29:**
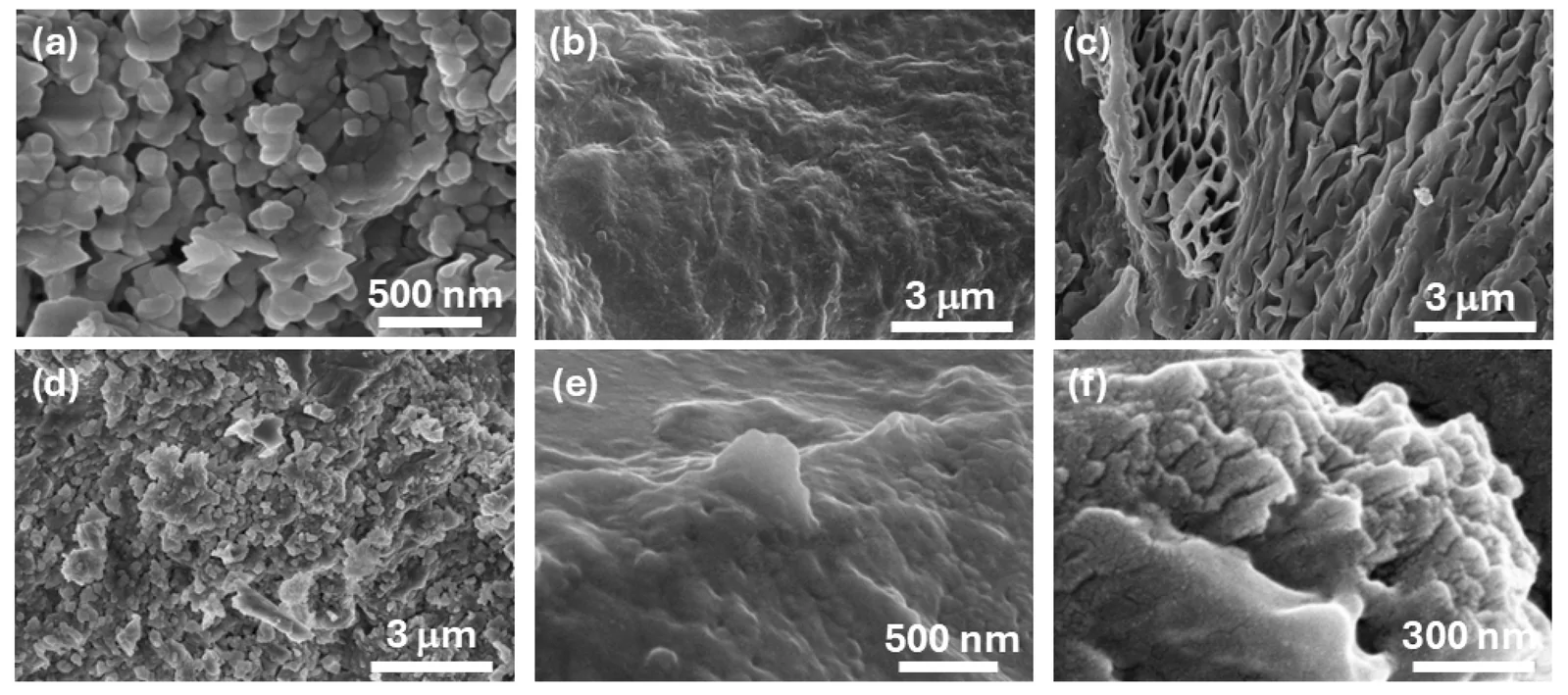
SEM images of PEGMEA/PEGDA polymer composite films containing (**a**) Ni2Co1MOF, (**b**) 3 wt.% rGO–Ni2Co1MOF, (**c**) 10 wt.% rGO–Ni2Co1MOF, (**d**) Ni1Co2MOF, (**e**) 3 wt.% rGO–Ni1Co2MOF, and (**f**) 10 wt.% rGO–Ni1Co2MOF, illustrating the surface morphology and the effects of the MOF composition and rGO loading on the morphologies of the polymer composites.

In the SEM image of the pure Ni2Co1MOF-added film (Figure 29a–c), one can see agglomerates consisting of particles of Ni-Co MOF of ~500 nm in size, being grouped non-homogeneously. Thus, the Ni-Co MOF particles in the composite film aggregate rather than being homogenously dispersed. As for the Ni1Co2-MOF-added film (Figure 29d–f), its SEM image demonstrates larger and heterogeneously grouped agglomerates in areas of ~3 µm. One can say that there are MOF compositions that are rich in Co that have been obtained with the help of larger crystals than in Ni-rich systems. Agglomeration reduces the charge transfer performance as well as thermal barrier effectiveness. Morphologically, this accounts for the poor thermal stability observed in the TGA. In the SEM image of the 3% rGO-added film (Figure 29b), one can observe the surface becoming flat and acquiring a wavy nature. Agglomeration is greatly reduced due to the positioning of MOF particles between or on the rGO surfaces. Thus, the expansion of the polymer–filler interface and an improvement in thermal barrier effectiveness occur. The SEM image of the 10% rGO-Ni2Co1MOF-added film (Figure 29c) illustrates a layered, porous, and network-like structure. One should note that rGO is layered and is arranged macroscopically. One can observe great relief and morphological proof of percolation network formation.

A similar pattern emerges when observing images of the films with 3% and 10% of rGO-Ni1Co2MOF additives (Figure 29e,f): 3% of rGO additive results in a homogeneous and moderately layered surface, whereas 10% additive is accompanied by the curling and layering of rGO sheets. The high-resolution image in the case of the 10% rGO-Ni1Co2MOF sample at the 300 nm level (Figure 29f) illustrates that the rGO sheets become curled and folded and come into contact with the Ni-Co MOF particles.

To summarize, one can state that rGO achieves a synergistic effect at a small 3% additive rate, acting as an effective carrier and bridge for MOF particles. This helped to optimize the thermal characteristics of the polymer to the fullest extent possible. However, at 10%, rGO stopped being a reinforcer and became a self-layered element, disrupting the integrity of the polymer, thus resulting in agglomeration. The vertical layers observed in Figure 29c can be regarded as evidence of this fact. Thus, the SEM results fully corroborate the observations made in TGA: 3% is the most stable. In this paper, the 3% rate of rGO addition may be referred to as the “threshold value” (percolation threshold) [76,77].

## 5. Conclusions

The preparation of bimetallic Ni-Co coordination framework materials with nominal molar Ni/Co ratios of 2:1 and 1:2 using a rapid, sub-100 °C, continuous-wave laser-induced synthesis method was demonstrated. It was shown that the metal ratio substantially influenced the structural, photophysical, and electrochemical properties of the obtained samples. In comparison to Ni1Co2MOF, Ni2Co1MOF possesses a higher BET surface area (88.3 m^2^/g vs. 52.5 m^2^/g) and total pore volume (0.022 cm^3^/g vs. 0.016 cm^3^/g), with their mean calculated BJH pore diameters being 3.38 and 3.04 nm, respectively. The coordination of carboxylate and nitrogen-donor ligands with the Ni and Co centers was observed spectroscopically. The oxidation states of the dominant species in the samples were Ni^2+^ and Co^2+^. However, it should be noted that the presence of reduced-metal components needs to be assessed by physically constrained XPS analysis. Furthermore, it can be seen that the Ni/Co ratio also affects the decay dynamics, implying different radiative and non-radiative relaxation mechanisms.

The introduction of rGO into the sample increased the charge transport efficiency due to the formation of electrically conductive paths and interfacial polarization. At a potential scan rate of 10 mV/s in an acidic electrolyte, the specific capacitance of the 10 wt.% rGO-Ni1Co2MOF composite was equal to 32.3 F/g and it maintained 98% of its capacity over 400 cycles; similarly, the corresponding Ni2Co1-based composite retained about 96% of its response. The electrochemical performance of the sample included both diffusion control and pseudocapacitive effects.

In UV-cured PEGMEA/PEGDA samples containing rGO, the dependence of the electrical and dielectric properties on the frequency and rGO content was observed. Moderate rGO incorporation favors the formation of conductive networks and a Maxwell–Wagner–Sillars interfacial polarization effect, while its excess leads to aggregation and a decrease in the beneficial effect caused by the conductive phase.

The main result of this work is the realization of a rapid, sub-100 °C laser-assisted preparation method for Ni-Co coordination framework/rGO materials and the relation of the Ni/Co ratio and rGO content to the material’s pore structure, charge transport properties, and dielectric response. However, it is necessary to perform high-resolution XPS analyses for the more accurate assessment of phase purity and minor metallic species, as well as to characterize the excited state and electrochemical properties.

## Figures and Tables

**Table 1:** The isotherm results of the NiCoMOFs (2:1–1:2).

Sample	BET Surface Area	Total Pore Volume	BJH Pore Radius	Mean Pore Diameter
Ni2Co1MOF	88.3 m^2^ g^−1^	0.022 cm^3^ g^−1^	1.69 nm	3.38 nm
Ni1Co2-MOF	52.5 m^2^ g^−1^	0.016 cm^3^ g^−1^	1.52 nm	3.04 nm

## Data Availability

The original contributions presented in this study are included in the article and Supplementary Materials. Further inquiries can be directed to the corresponding authors.

## Supplementary Materials

The following supporting information can be downloaded at: https://www.mdpi.com/article/10.3390/polym18161985/s1. Figure S1: Deposition of NiCoMOF (2:1–1:2) and (1–10%) rGO-NiCoMOF (2:1–1:2) composites onto ITO glass surface by drop method.; Figure S2: Preparation of PEGMEA/PEGDA Thin Films Containing a Pho-toinitiator and MOFs by In Situ Photopolymerization; Figure S3: SEM images of (a) Ni2Co1MOF and (b) Ni1Co2MOF containing 10% rGO.
