# Supplementary material for: Synthesis of Ni-Co Metal–Organic Framework (Ni-Co MOF) Structures by High-Power, Continuous Laser-Induced Rapid Synthesis Method and Investigation of Their Morphological, Structural, Photophysical, and Electrical Properties
Source: Polymers (Basel). 2026 Aug 14;18(16):1985. doi: 10.3390/polym18161985 (PMC13516495; doi:10.3390/polym18161985)
Supplement: Supplementary file 1 [file polymers-18-01985-s001.zip › polymers-4439810-supplementary.pdf]

Supporting Information

Synthesis of Ni-Co Metal–Organic Framework (Ni-Co MOF) Structures by High-Power, Continuous Laser-Induced Rapid Synthesis Method and Investigation of Their Morphological, Structural, Photophysical, and Electrical Properties

Saliha Mutlu [1,2], Bülend Ortaç [2,*], Ali Karatutlu [3], Vildan Yılmaz [4], Süreyya Aydin Yüksel [4], Ahmet Hakan Yilmaz [5], Nergis Arsu [6,*] and Sevil Savaskan Yilmaz [1,2,*]

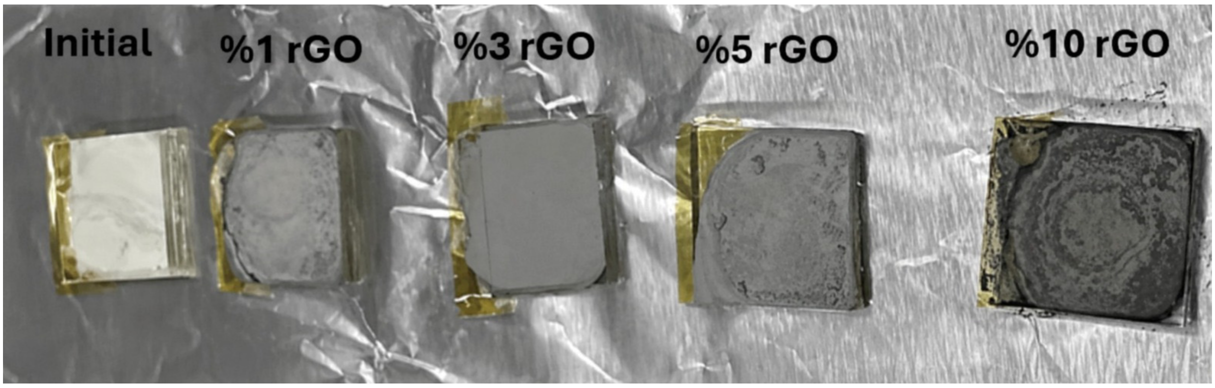

Figure S1. Deposition of NiCoMOF (2:1-1:2) and (1-10%) rGO-NiCoMOF (2:1-1:2) composites onto ITO glass surface by drop method.

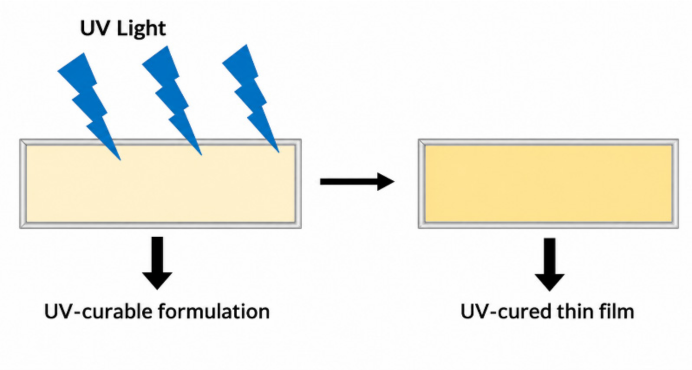

Figure S2. Preparation of PEGMEA/PEGDA Thin Films Containing a Photoinitiator and MOFs by *In Situ* Photopolymerization

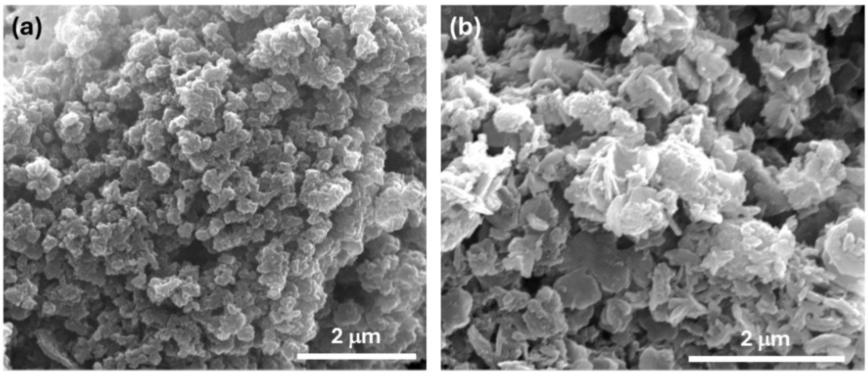

Figure S3. SEM images of (a) Ni2Co1MOF and (b) Ni1Co2MOF containing 10% rGO.
